# Trade-Offs Among Arc Erosion Resistance, Wear Resistance, and Compressive Performance: Designing Cu-Nb-Gr Composites with a Semi-Continuous Gr-Rich Structure Coupled with an Nb-Rich Load-Bearing Structure

**DOI:** 10.3390/ma19163429

**Published:** 2026-08-13

**Authors:** Qingchuan Zhan, Yong Li, Zhe Wang, Yin Zhang, Xiaohui Zhao, Cheng Fang, Junshan Fan, Xuegui Hu

**Affiliations:** 1 School of Materials Science and Engineering, Xi’an Polytechnic University, Xi’an 710048, China; 18752166541@163.com (Q.Z.); 15028319674@163.com (J.F.); 2 Hunan Jiatong New Materials Co., Ltd., Changde 415000, China; ct3ing@gmail.com (Y.L.); 15375692061@163.com (X.H.); 3 Changde Jiahui Hydraulic Machinery Co., Ltd., Changde 415000, China; 4 School of Physics, Xi’an Jiaotong University, Xi’an 710049, China; 17667819441@163.com

**Keywords:** Cu-Nb-Gr composites, Gr regulation mechanism, semi-continuous structure, property balance

## Abstract

Developing Cu-based composites with excellent electrical and mechanical properties under multiphysics-coupled service conditions remains challenging. Novel Cu-Nb-Gr composites were fabricated by high-energy ball milling and High-pressure Multi-field Assisted Rapid Sintering. Experiments combined with computational fluid dynamics (CFD) and finite element method (FEM) simulations were used to investigate how Gr regulates material performance. The incorporation of 3 vol.% Gr promoted the formation of a semi-continuous Gr-rich structure coupled with an Nb-rich load-bearing structure. Under arc erosion, the semi-continuous Gr-rich structure provided efficient heat-conduction pathways, reducing the peak temperature and metal-vapor recoil force, while the Nb-rich load-bearing structure suppressed liquid–metal spattering and stabilized the molten pool. Simultaneously, Gr dynamically spread to form a continuous solid-lubricating film during sliding friction, significantly reducing the coefficient of friction and interfacial shear stress. Furthermore, under compressive loading, the semi-continuous Gr-rich structure coupled with the Nb-rich load-bearing structure alleviated interfacial elastic–modulus mismatch and extreme stress concentration, limiting macroscopic plastic deformation of the matrix. Consequently, Cu-Nb-3Gr achieved a favorable balance of arc-erosion resistance, wear resistance, and compressive performance, providing a new strategy for improving conventional Cu-based composites.

## 1. Introduction

In aerospace, deep-sea and offshore engineering, and specialized equipment applications, core components must operate reliably over long periods under multiphysics-coupled service conditions [1,2,3,4]. For such core components, Cu-based composites must withstand high-frequency mechanical compression and cyclic impact over extended periods [5,6,7], provide excellent friction and wear resistance under sliding contact [8,9,10], and resist severe erosion from high-voltage arc discharges and thermal shocks [11,12,13]. Accordingly, the next generation of high-performance Cu-based materials must combine high strength and toughness, a low coefficient of friction, and outstanding thermal erosion resistance while retaining high electrical and thermal conductivities [14,15,16,17]. Although conventional Cu-based composite systems, including Cu-Mg [18], Cu-Ag [19], and Cu-Nb [20], have achieved major advances in balancing strength and toughness, electrical and thermal conductivity, friction reduction, and wear resistance, their ability to ensure long-term safe operation under harsh high-temperature and high-pressure conditions remains uncertain. Therefore, exploring novel Cu-based composite systems that combine high strength, high conductivity, and high tolerance to multiphysics-coupled environments is of considerable importance.

Among numerous binary Cu-based systems, Cu-Nb composites prepared through in situ routes or powder metallurgy have emerged as a major research focus in the field of metal-matrix composites because of their distinctive combination of high strength and high conductivity [21,22,23]. Nb has a high melting point of 2468 °C, high stiffness, and low solid solubility in the Cu matrix. During bulk consolidation, the Nb phase promotes the formation of an Nb-rich load-bearing structure within the soft Cu matrix. Without significantly impairing electron transport through the matrix, this load-bearing structure acts as an array of “load-bearing columns” that constrains plastic flow and creep of the matrix [24,25]. For example, A. Spirin et al. [26] employed in situ sintering to prepare Cu-Nb composites and found that the fine grains of the constituent phases and high grain-boundary density confer greater strength and thermal stability. Tang et al. [27] compared Cu-Nb with pure Cu and found that the introduction of Nb effectively improves erosion resistance, with the Cu-Nb material exhibiting smaller erosion-pit volumes and depths. However, conventional binary Cu-Nb composites still exhibit relatively high interfacial friction resistance under high-speed and high-load sliding wear [28,29]. Moreover, the absence of an effective solid lubricant and additional heat-dissipation pathways for transient heat flux renders the near-surface molten pool susceptible to instability and boiling-induced metal spattering under repeated arc bombardment. These processes increase surface roughness and trigger premature fatigue cracking [30,31], severely limiting the service reliability of this material system under complex conditions [32,33].

To further improve the performance of Cu-Nb composites, introducing a third phase to establish a synergistic strengthening mechanism has emerged as a promising strategy [34,35]. For example, Tang et al. [36] found that adding an appropriate amount of Ti to the Cu-Nb system modifies the crystal structure of the Nb phase and promotes the formation of interphase TiO, thereby alleviating stress concentrations at the Cu/Nb interface and increasing the strength of the Cu-Nb material. Nevertheless, this strategy cannot fundamentally overcome the performance limitations of the binary system under friction and arc erosion. Graphene (Gr), a two-dimensional carbon nanomaterial, represents an ideal solid lubricant and thermally conductive reinforcement because of its low interlayer shear strength and high intrinsic thermal conductivity [37,38,39]. For example, Li et al. [40] experimentally demonstrated that incorporating Gr into a Cu matrix fully exploits its solid-lubrication effect and markedly reduces the dynamic coefficient of friction. Yu et al. [41] used cold rolling to fabricate Cu-Gr materials and demonstrated that these composites exhibit excellent mechanical–electrical synergy and outperform pure Cu at elevated temperatures. In addition, Gr acts as a barrier to dislocation rearrangement and relaxation during heat treatment, further improving high-temperature stability. Incorporating Gr into the Cu-Nb system can therefore provide continuous interfacial self-lubrication during sliding [42,43] and promote the formation of a semi-continuous Gr-rich heat-conduction structure within the matrix to withstand extreme arc-induced thermal shocks [44], thereby enhancing comprehensive performance under complex service conditions.

Although extensive experiments have confirmed that the incorporation of Gr effectively improves the comprehensive performance of Cu-based composites, failure under complex conditions such as arc erosion and sliding friction often involves transient evolution over extremely short timescales. Conventional experiments provide limited access to these transient changes and their underlying mechanisms. Recent studies indicate that conventional experimental characterization typically captures only static residual morphologies after material failure and solidification, providing insufficient information about transient physical processes such as liquid-metal spattering under extreme heat fluxes. To overcome the limitations of static observations, multiphysics numerical simulations integrating CFD and three-dimensional FEM have become an effective approach for investigating microstructure-property relationships in multiphase materials [45,46]. For example, Cheng et al. [47] used a CFD model to investigate the mechanisms governing multiphase flow, heat transfer, mass transport, melting, and solidification in a Cu-Ni system, thereby confirming the reliability of CFD for resolving thermomechanically coupled failure in composites. Chen et al. [48] employed FEM to quantify the influence of material type on fatigue crack propagation (FCP) and damage accumulation (DA) behavior. However, these studies treated CFD and FEM separately and therefore provided no comprehensive interpretation of material performance. More recently, Fang et al. [49] from our group combined CFD and FEM to elucidate the synergistic effects of microstructure on the arc erosion resistance and mechanical properties of Ag-based contacts, providing a new route for material design. Nevertheless, multiphysics numerical studies that clarify the intrinsic relationship between multiphase microstructure, particularly the spatial distribution of Gr, and material performance remain scarce for Cu-Nb-Gr ternary composites.

Accordingly, Cu-Nb-Gr composites with a novel semi-continuous structure were fabricated through high-energy ball milling followed by H-MARS. To comprehensively investigate arc erosion evolution, friction and wear behavior, and compression resistance under service conditions, experiments were combined with multiphysics simulations. Three-dimensional models of the composites were reconstructed using phase-identification techniques. CFD was employed to examine the evolution of the molten pool and molten bridge during arc erosion, while arc erosion experiments were used to evaluate the influence of the Gr microstructure on arc erosion resistance. Furthermore, FEM simulations were conducted based on experimental data to characterize the stress and deformation distributions on the material surfaces under frictional wear and mechanical impact. This study not only elucidates the mechanism by which an appropriate Gr content promotes the formation of a semi-continuous Gr-rich structure coupled with an Nb-rich load-bearing structure within the matrix but also clarifies the regulatory role of this semi-continuous structure in arc erosion resistance, wear resistance, and compression resistance. The findings provide a new strategy for developing Cu-based composites with a favorable balance among arc erosion resistance, wear resistance, and compression resistance.

## 2. Materials and Methods

### 2.1. Material Preparation

#### 2.1.1. Preparation of Cu-Nb-Gr Composite Powders

This study adopted an integrated experimental and computational approach to systematically investigate the microstructure, dynamic arc erosion behavior, and mechanical properties of the Cu-Nb-1Gr, Cu-Nb-3Gr, and Cu-Nb-5Gr composites. The raw materials included high-purity Cu powder (Kangshuo Intelligent Manufacturing Co., Ltd., Yingtan, China), Nb powder (Guangzhou Metal Metallurgy Co., Ltd., Guangzhou, China), and single-layer Gr powder (Hanzhuo Metal Materials, Baoding, China). The Cu powder had a purity of 99.9 wt.%, an average particle size of approximately 5 μm, and a density of 8.96 g cm^−3^. The Nb powder had a purity of 99.9 wt.%, an average particle size of approximately 3 μm, and a density of 8.57 g cm^−3^.

Owing to the extremely high specific surface area of Gr and its poor wettability with metals, macroscopic agglomeration readily occurs as the Gr content increases. Under mechanical impact and compressive stress, severe microscopic phase agglomeration produces highly nonuniform strain partitioning and extreme interfacial stress concentrations within local microregions. These effects induce microcrack initiation and propagation at an early stage of loading, ultimately causing premature structural collapse or brittle fracture. Therefore, precise control of the volume fraction of Gr is essential for improving the comprehensive performance of this composite system.

To prepare homogeneous composite powder mixtures, Cu powder, Nb powder, and Gr powder at different volume fractions were proportionally mixed and subjected to mechanical alloying in a high-energy planetary ball mill (MSK-SFM-3-II, Beijing Jinyang Wanda Technology Co., Ltd., Beijing, China). The content of Nb was maintained at 10 vol.% in all three samples, while the volume fractions of Gr were set to 1, 3, and 5 vol.%. Ball milling was conducted at room temperature under an inert Ar atmosphere. Anhydrous ethanol served as the process control agent, and the ball-to-powder mass ratio was set to 20:1. The rotational speed was set to 400 r min^−1^, and the powders were ball-milled for 3 h, yielding three Cu-Nb-Gr composite powder mixtures with different Gr contents.

#### 2.1.2. Preparation of Bulk Cu-Nb-Gr Composites

Following high-energy ball milling, the powder mixtures were annealed in a vacuum drying oven at 673 K for 1.5 h to relieve work hardening and evaporate the residual solvent. The powders were subsequently cold-pressed at room temperature into cylindrical green compacts with a diameter of 35 mm. The green compacts were placed in a high-strength graphite die and consolidated in a High-pressure Multi-field Assisted Rapid Sintering furnace (H-MARS-500T-300, JIATONG, Shanghai, China). The sintering schedule was set as follows. The compacts were first heated to 780 K at 50 K min^−1^ and held for 5 min. The temperature was then increased to 1173 K at 30 K min^−1^, and the compacts were held for 10 min. A constant axial pressure of 90 MPa was applied and maintained throughout the sintering cycle. After cooling, the dense bulk composites were removed from the die. For subsequent characterization and discussion, the composites containing 1, 3, and 5 vol.% Gr were designated Cu-Nb-1Gr, Cu-Nb-3Gr, and Cu-Nb-5Gr, respectively.

### 2.2. Phase and Microstructural Characterization

XRD (Shimadzu Ltd., Kyoto, Japan) was employed to analyze the phase composition and crystal-structure evolution of the composites before and after sintering. Measurements were performed using Cu Kα radiation (λ = 1.54060 Å) at an operating voltage of 40 kV and a current of 40 mA. FE-SEM (JEOL JSM-7000F, (JEOL Ltd., Tokyo, Japan) was used for high-resolution characterization of the powder morphology after ball milling, the microstructure of the bulk composites after sintering, the surface morphologies after wear, and the morphologies of erosion pits after vacuum arc breakdown.

### 2.3. Vacuum Arc Breakdown Testing

**Figure 1:**
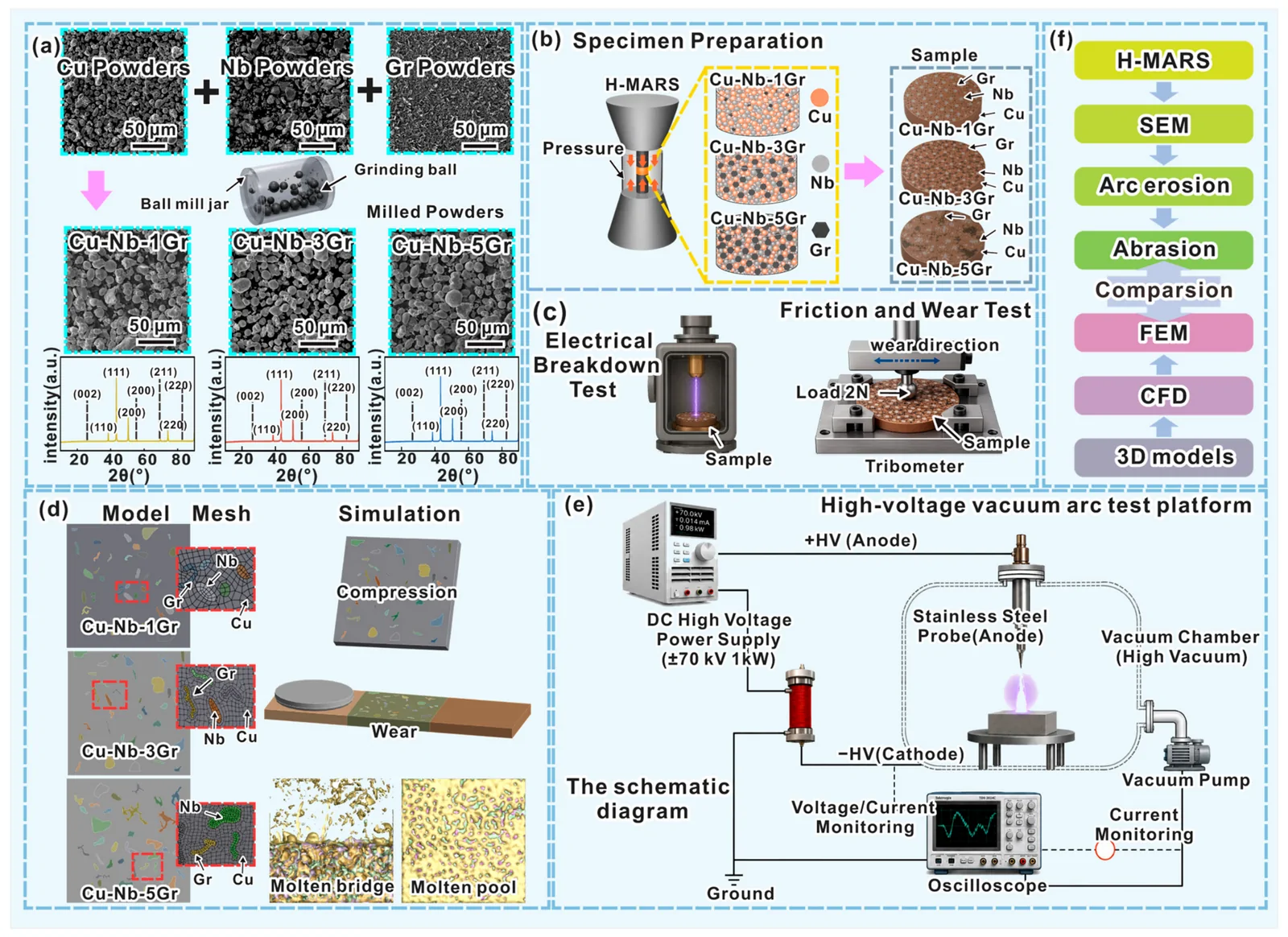
Schematic illustration of the experimental and numerical simulation procedures: (**a**) initial morphologies of the Cu, Nb, and Gr powders; (**b**) sample preparation via FHPS; (**c**) electrical breakdown testing and friction-and-wear testing; (**d**) mesh generation and three-dimensional models for CFD and FEM simulations; (**e**) schematic diagram of the in-house high-voltage vacuum arc test platform; and (**f**) overall research workflow.

The voltage-breakdown characteristics of the materials were evaluated using an in-house ultra-high-voltage vacuum arc test platform. Figure 1e presents the corresponding circuit schematic. The platform incorporated a DC high-voltage power supply with a maximum output voltage of ±70 kV, a rated power of 1 kW, and a maximum current of approximately 80 A. Before testing, the bulk samples were ground, polished, and fixed on the sample stage as the cathode. The glass test chamber was evacuated to a vacuum level of 2.5 × 10^−4^ Pa to minimize material oxidation during the high-temperature self-breakdown process. A stainless-steel probe served as the anode, and the initial gap between the probe tip and sample surface was precisely set to 1 cm. No fixed breakdown voltage was preset for individual events. The voltage was gradually increased by adjusting the control knob of the DC high-voltage power supply until an arc was initiated at the tip of the stainless-steel probe. The arc then propagated to the sample surface, completing a single discharge event. Each of the three composite samples was subjected to 50 consecutive breakdown events at the same surface location to systematically investigate the effects of the Gr content on the arc erosion morphology and dielectric strength. The discharge duration and deposited energy of individual breakdown events were not independently monitored. Accordingly, the arc erosion results are interpreted as relative comparisons among the three composites tested under identical nominal conditions.

### 2.4. Dry Sliding Friction and Wear Testing

The tribological properties of the Cu-Nb-1Gr, Cu-Nb-3Gr, and Cu-Nb-5Gr composites were evaluated under dry sliding conditions at room temperature using a planar friction tester (ZJ-339-GSR, Shenzhen Zhijia Instrument Co., Ltd., Shenzhen, China). To reproduce the contact configuration of the tribopair, a GCr15 bearing-steel ball or a stainless-steel ball with a diameter of 9.525 ± 0.05 mm was selected as the counterbody. Before testing, the surfaces of all bulk samples were sequentially ground with SiC abrasive papers and mechanically polished to a mirror finish using diamond paste. The samples were subsequently ultrasonically cleaned in anhydrous ethanol for 10 min to completely remove residual surface contaminants. Based on preliminary experiments and the load-bearing characteristics of the materials, the normal load, sliding velocity, and duration of each test were set to 2 N, 20 mm s^−1^, and 700 s, respectively.

During testing, the force sensor and data-acquisition system of the instrument continuously recorded the COF at a high sampling rate and generated the COF curves in real time. At least three replicate tests were conducted for each composite under identical conditions to ensure experimental repeatability and statistical reliability. After testing, the wear tracks on the sample surfaces were observed and imaged using an optical metallographic microscope (Axio Scope, Beijing Precise Instrument Co., Ltd., Beijing, China). The degree of wear of each sample group was evaluated by measuring the two-dimensional morphology and width of each wear track. The friction and wear mechanisms of the composites were further investigated by correlating these measurements with the damage features on the surfaces after wear, including plowing grooves and spalling.

### 2.5. CFD and FEM Simulations

Based on the actual microstructural features, local three-dimensional representative volume element (RVE) models of the Cu-Nb-1Gr, Cu-Nb-3Gr, and Cu-Nb-5Gr composites, with dimensions of 200 × 200 × 100 μm^3^, were constructed using SolidWorks. The models were imported into ANSYS Meshing for mesh generation and subsequently transferred to ANSYS Fluent for dynamic arc erosion simulations. The VOF multiphase-flow model, k-ε turbulence model, melting/solidification model, and MHD module were coupled in the simulations. The SIMPLE algorithm was employed, with a time step of 0.001 s and a maximum of 100 iterations per time step, to simulate the dynamic evolution of the molten pool and material erosion under more than 10^4^ thermal cycles.

To further investigate the mechanical response of the materials after erosion, a three-dimensional mechanical model of the surface layer after erosion was reconstructed using surface data from CFD, MATLAB-based visual recognition, and SolidWorks. The procedure comprised the following steps. First, the morphological images were preprocessed to enhance the contrast. Next, in-house code was used for image segmentation and microstructural feature extraction. Finally, the in-house code generated the geometric features. These features were imported into SolidWorks to construct the three-dimensional solid model and subsequently transferred to ANSYS Workbench for structural FEM analysis.

During FEM mesh generation, the mesh size at the structural boundaries was defined as 0.02 mm, and a Fixed Support was applied to the bottom of the model to reproduce the actual boundary conditions. In the Static Structural simulation, a load was applied to the top surface of the sample to evaluate the compressive strength. The pressure was linearly increased to 27.6 MPa from 0 to 0.2 s, maintained at 27.6 MPa from 0.2 to 0.3 s, and subsequently reduced to 0 MPa from 0.3 to 0.5 s. In the Transient Structural simulation of sliding friction and wear, the sliding velocity and normal load were set to 10 mm s^−1^ and 3000 N, respectively. To represent the actual material properties, the COF between the slider and Cu matrix was set to 0.2, whereas the COF between the slider and Gr or the solid-lubricant transfer film was set to 0.1. Interfacial contacts among the constituent phases were defined as bonded to represent sound metallurgical bonding, whereas the contact between the friction counterparts was defined as frictional. The adaptive time-step range of the solver was set from 0.001 to 0.01 s to ensure convergence and numerical accuracy. Figure 1 presents the overall experimental and simulation workflow, while Table 1 summarizes the material properties, boundary conditions, and model simplification assumptions.

## 3. Results and Discussion

### 3.1. Micromorphological Characterization of Cu-Nb-Gr Materials

**Figure 2:**
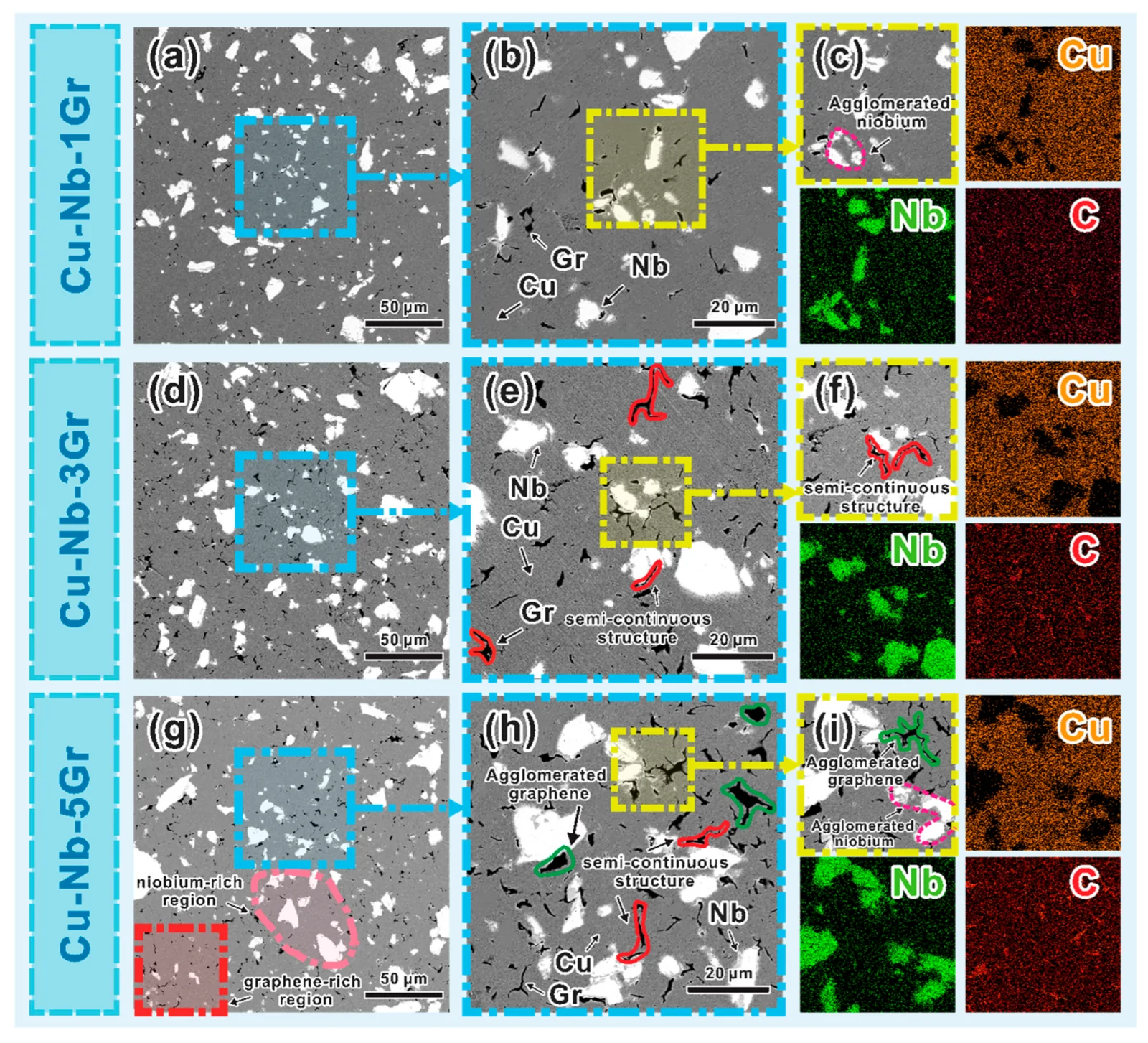
Micromorphologies and EDS mappings of the sintered materials. (**a**–**c**) Cu-Nb-Gr. (**d**–**f**) Cu-Nb-3Gr, highlighting the semi-continuous structure. (**g**–**i**) Cu-Nb-5Gr, showing pronounced agglomeration of Gr and Nb.

The microstructures of the samples were characterized by SEM, as shown in Figure 2a,d,g. Figure 2a presents the micromorphology of the Cu-Nb-1Gr sample containing 1 vol.% Gr. Based on the EDS mappings in Figure 2c, the gray, white, and black regions are identified as the Cu matrix, Nb phase, and Gr phase, respectively. Figure 2a shows that the Nb phase is distributed relatively uniformly throughout the Cu matrix, with an average particle size of approximately 8 μm. By contrast, the Gr phase is dispersed throughout the Cu matrix as fine, discrete spots.

As the Gr content increases to 3 vol.% in Figure 2d, several Gr regions become clearly discernible even at low magnification. Notably, the number of Nb particles decreases, whereas the size of individual Nb particles increases markedly, with the maximum particle size reaching approximately 45 μm. When the Gr content further increases to 5 vol.% in Figure 2g, the distribution of the Nb phase becomes heterogeneous, and pronounced local Nb agglomeration is observed.

To further examine the micromorphologies of Nb and Gr, a magnified image of the central region of the Cu-Nb-1Gr sample is provided in Figure 2b. The Nb phase exhibits an isolated island-like distribution within the matrix. Moreover, the high-magnification SEM image of the yellow-boxed region in Figure 2c reveals partial agglomeration of the Nb phase, while a small amount of Gr is distributed around the Nb phase. Similarly, the central region of the Cu-Nb-3Gr sample is presented at higher magnification in Figure 2e. The Nb phase is uniformly distributed, and no evident agglomeration is observed. As Nb is characterized by a high melting point and high hardness, its uniform distribution throughout the matrix favors the formation of an Nb-rich load-bearing structure, which is expected to preserve the overall structural integrity of the material under high-temperature and high-pressure conditions. The enlarged image of a representative region in Figure 2e is presented in Figure 2f and reveals that increasing the Gr content to 3 vol.% induces slight local agglomeration, leading to the formation of a semi-continuous structure. Given the excellent intrinsic thermal conductivity of Gr, when the composite is exposed to the intense thermal environment generated by an electric arc, this semi-continuous Gr structure may rapidly transfer transient heat from localized regions throughout the matrix via in-plane thermal conduction, thereby mitigating surface damage associated with localized heat accumulation. Figure 2f also shows that some Gr regions tend to surround the Nb phase. Gr may therefore serve as a bridging phase and facilitate the formation of a semi-continuous Gr-rich heat-conduction structure coupled with an Nb-rich load-bearing structure, which is expected to further enhance the overall performance of the composite.

Because local Nb-rich and Gr-rich regions occur in the Cu-Nb-5Gr sample, a region displaying comparatively uniform distributions of Nb and Gr at low magnification was selected for closer examination, as shown in Figure 2h. Nevertheless, the high-magnification image reveals severe Gr agglomeration, and the corresponding regions are marked by green circles. Such agglomeration may compromise the semi-continuous Gr-rich heat-conduction structure and promote localized overheating at elevated temperatures, thereby significantly shortening the service life of the material. Moreover, severe Gr agglomeration occupies regions that would otherwise accommodate the Nb phase, indirectly disrupting the Nb-rich load-bearing structure and potentially weakening the mechanical properties of the composite.

Overall, at a Gr content of 3 vol.%, the more homogeneous distributions of the Nb and Gr phases are expected to promote the formation of an Nb-rich load-bearing structure and a semi-continuous Gr-rich heat-conduction structure, thereby maintaining the stability of the material under extreme conditions involving high temperatures and pressures. By contrast, a further increase in the Gr content induces pronounced Gr agglomeration and a heterogeneous Nb distribution, potentially disrupting the semi-continuous Gr-rich heat-conduction structure and Nb-rich load-bearing structure within the matrix and consequently degrading the electrical and mechanical properties. To verify these hypotheses, the electrical and mechanical properties will be systematically investigated through combined experimental and simulation approaches.

Table 2 lists the electrical conductivity, hardness, and density of the Cu-Nb-1Gr, Cu-Nb-3Gr, and Cu-Nb-5Gr samples. The Cu-Nb-3Gr sample exhibits higher hardness without appreciably compromising its electrical conductivity. Although the hardness of the Cu-Nb-5Gr sample also increases to some extent, its electrical conductivity decreases markedly. The contrasting behavior may be attributed to the homogeneous semi-continuous structure in Cu-Nb-3Gr, which synergistically reinforces the matrix while preserving the continuity of electron-transport pathways within the Cu matrix, whereas severe phase agglomeration in Cu-Nb-5Gr increases electrical resistance and consequently impairs electrical conductivity.

### 3.2. Arc-Eroded Surface Morphologies of the Cu-Nb-1Gr, Cu-Nb-3Gr, and Cu-Nb-5Gr Materials

**Figure 3:**
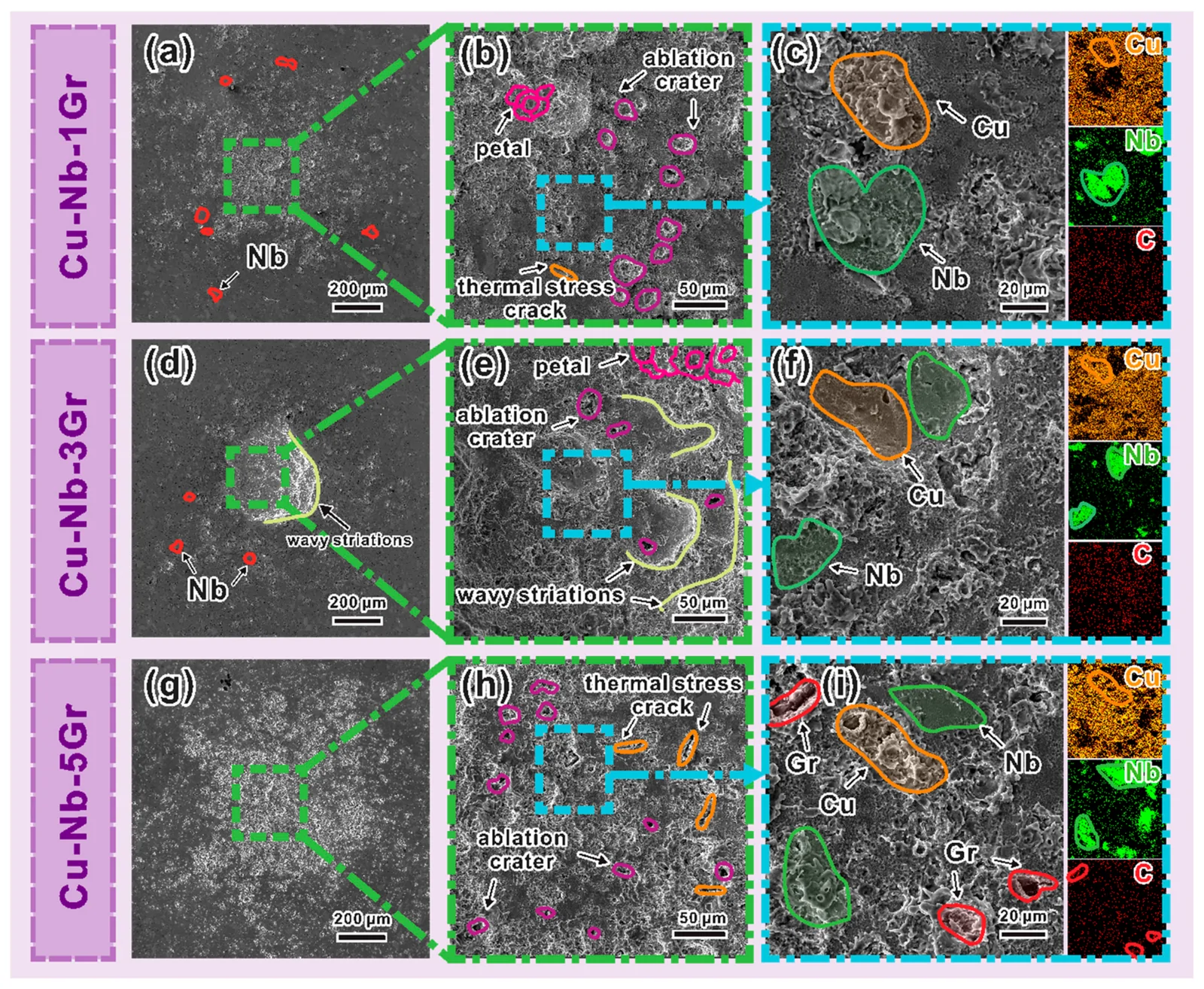
Surface morphologies and EDS mappings of the materials following 50 vacuum arc breakdown tests. (**a**–**c**) Cu-Nb-1Gr, showing thermal-stress cracks and erosion craters. (**d**–**f**) Cu-Nb-3Gr, showing wavy striations and mild erosion. (**g**–**i**) Cu-Nb-5Gr, showing widespread diffuse erosion damage and deep thermal-stress cracks caused by Gr agglomeration.

Building on the detailed characterization of the initial microstructures of the three bulk composites, electrical breakdown tests were conducted on all three samples under vacuum to further elucidate the influence of Gr content on the arc erosion resistance under practical breakdown conditions, and the post-test surface micromorphologies were subsequently characterized systematically by SEM, as shown in Figure 3a,d,g. After 50 electrical breakdown events, the central region of the Cu-Nb-1Gr sample in Figure 3a exhibits network-like erosion damage extending radially outward. Notably, the propagation paths of the damaged regions show a pronounced tendency to bypass the Nb phase. After the same number of breakdown events, the surface of the Cu-Nb-3Gr sample in Figure 3d remains largely intact, except for a localized erosion crater at the center. This enhanced resistance may be attributed to the formation of a semi-continuous Gr-rich heat-conduction structure at a Gr content of 3 vol.%, which enhances the effective thermal conductivity of the composite. When the arc heat flux impinges on the surface, the resulting thermal energy can be rapidly transported along the highly conductive Gr pathways into the surrounding cooler matrix and deeper regions, thereby markedly reducing the peak temperature at the arc root. In addition, bright-white wavy solidification striations are observed at the center of the sample. However, as the Gr content further increases to 5 vol.%, the surface of the Cu-Nb-5Gr sample in Figure 3g exhibits widespread and diffuse erosion damage. Based on the microscopic structural features in Figure 2h, this extensive damage is primarily attributed to the disruption of the semi-continuous Gr-rich heat-conduction structure by Gr agglomerates, which increases the local thermal resistance and impedes the conduction and dissipation of arc heat, ultimately producing severe thermal erosion over a large surface area.

High-magnification examination of the breakdown centers reveals that the Cu-Nb-1Gr surface in Figure 3b contains a dense sequence of erosion craters and a small number of thermal-stress cracks. A typical petal-like morphology is also observed on the surface. This feature is identified as a metallic melt-resolidified layer that develops through melting of the Cu matrix under a high-temperature arc and subsequent rapid cooling and solidification during arc extinction. By comparison, the number of erosion craters on the Cu-Nb-3Gr surface in Figure 3e decreases markedly, their distribution becomes highly discrete, and no evident thermal-stress cracks are observed. The bright-white wavy striations at the erosion center are also resolved more clearly in this image and represent the characteristic morphology of a shallow surface layer of Cu that melts at high temperature and subsequently resolidifies. The Cu-Nb-5Gr sample in Figure 3h exhibits the most severe arc erosion, with a sharp increase in both the number and size of the erosion craters and damage extending across nearly the entire region. The surface damage comprises an interwoven network of deep erosion craters and wide thermal-stress cracks, further demonstrating that failure of the semi-continuous Gr-rich heat-conduction structure triggers runaway, “avalanche-like” accumulation of local heat and ultimately causes extensive thermomechanical damage.

To elucidate the surface-damage mechanism after electrical breakdown and clarify the compositional evolution of the distinctive microstructures, representative regions in Figure 3b,e,h were selected for further magnification, and detailed elemental analyses were performed using EDS mappings. The results show that the spatial distribution of the petal-like structure on the Cu-Nb-1Gr surface, outlined in orange in Figure 3c, coincides closely with the distribution of Cu in the EDS mappings, providing strong evidence that this feature originates from melting and resolidification of the Cu matrix. Because the arc during vacuum electrical breakdown can generate temperatures that readily exceed the melting and boiling points of Cu, whereas the corresponding melting and boiling points of Nb are substantially higher, the Cu matrix melts preferentially and may even undergo localized vaporization under the same heat-flux loading, ultimately producing erosion craters and a surrounding melt-resolidified layer upon cooling. In addition, the Nb phase outlined in green in Figure 3c remains largely intact after breakdown, with only slight melting along its edges. This observation indicates that, at a low Gr content and in the absence of an effective Gr-rich heat-conduction structure, heat accumulation may raise the local temperature to a level approaching the melting point of Nb, resulting in thermal erosion along the Nb edges.

At a Gr content of 3 vol.% in Figure 3f, the high-magnification image shows that the portion of the Cu matrix outlined in orange remains relatively intact, and no evident signs of melting are observed in the Nb phase outlined in green. Together with the microscopic structural features in Figure 2e, these observations provide compelling evidence that the semi-continuous Gr-rich structure serves as an efficient pathway for heat spreading. The arc heat is rapidly redistributed, maintaining the local peak temperature below the melting point of Nb and mitigating the melting of the Cu matrix. The slight erosion craters under this condition mainly originate from relatively mild melting within a shallow surface layer.

However, on the Cu-Nb-5Gr surface in Figure 3i, deep Cu-dominated erosion craters outlined in orange are densely distributed, while even the refractory Nb phase undergoes severe bulk melting in the lower region outlined in green. Only the larger Nb-rich agglomerate in the upper region outlined in green retains a recognizable outline because of its larger volume. Combined with Figure 2h, these features indicate that severe Gr agglomeration interrupts the heat-conduction pathways through the matrix, increasing the local thermal resistance and causing substantial heat accumulation, ultimately leading to the boiling and splashing of Cu and extensive melting of Nb. Further examination reveals that the spatial locations of the large thermal-stress cracks and deep erosion craters in Figure 3i coincide closely with the Gr signals in the EDS mappings, indicating that the Gr agglomerates act as the primary initiation sites for destructive failure. On the one hand, the high interfacial thermal resistance within the agglomerated regions promotes the localization of arc heat, while the resulting high temperature drives vaporization deeper into the material, thereby producing large, deep erosion craters. On the other hand, the mismatch in the coefficient of thermal expansion (CTE) between the Gr agglomerates and the surrounding Cu matrix causes high thermal stresses to accumulate at the interfaces during instantaneous arc-induced thermal shock and rapid cooling after arc extinction, and the resulting residual thermal stresses ultimately fracture the matrix, inducing the initiation and catastrophic propagation of macroscopic thermal-stress cracks.

### 3.3. Dynamic Evolution of Molten Bridges and Molten Pools

**Figure 4:**
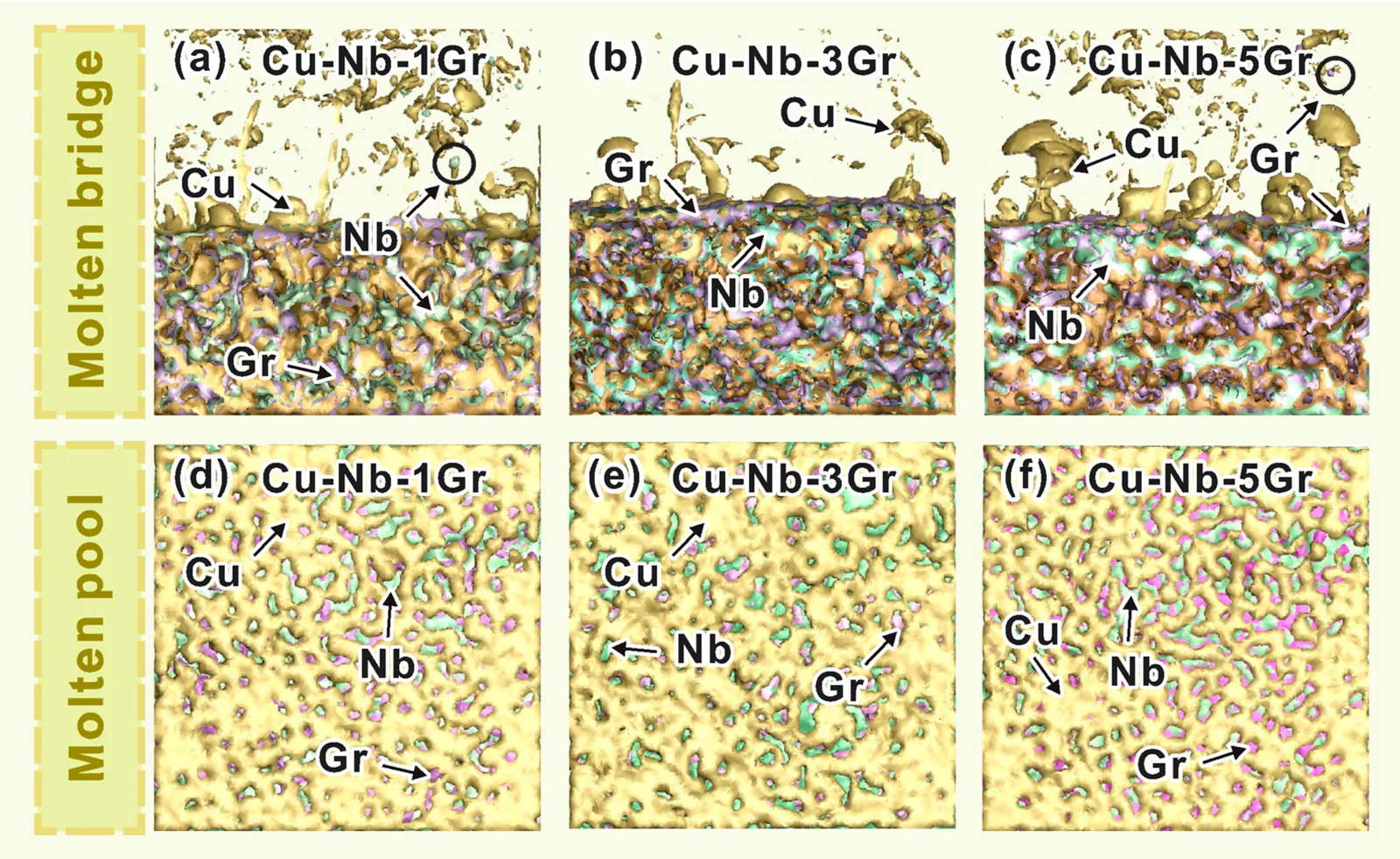
CFD simulation results for transient fluid dynamics during arc erosion. (**a**–**c**) Dynamic evolution of molten bridges in Cu-Nb-1Gr, Cu-Nb-3Gr, and Cu-Nb-5Gr. (**d**–**f**) Top views of the molten pools in the three samples.

Although the static SEM-based morphological characterization described above intuitively reveals the arc erosion outcomes at different Gr contents, static observations alone cannot resolve ultrafast transient fluid-dynamic processes, including the evolution of metal-vapor recoil force, liquid-metal splashing, and the flow-blocking effect of the Nb-rich load-bearing structure. Therefore, CFD/MHD simulations were further performed to analyze the evolution of the molten bridge at the instant of contact separation during arc erosion and the subsequent dynamic morphological changes in the molten pool, with the results presented in Figure 4.

Figure 4a–c present the simulated evolution of molten bridges in the three materials. Numerous fine molten Cu droplets are densely distributed above the Cu-Nb-1Gr surface. This response mainly arises because the low Gr content fails to establish an effective semi-continuous Gr-rich heat-conduction structure, allowing current constriction to generate intense Joule heat that accumulates rapidly within the necking region and causes the liquid Cu to overheat and undergo explosive vaporization. The resulting internal vapor pressure not only drives rapid fracture of the molten bridge but also generates a strong vapor recoil force. In the absence of effective support from an Nb-rich load-bearing structure, the low-viscosity liquid Cu is propelled through the molten-pool surface by the vapor recoil force and violently ejected outward. These simulated flow features provide strong mechanistic support for the formation of the peripheral petal-like melt-resolidified layer and deep erosion craters observed in the SEM image in Figure 3c.

By contrast, the molten-bridge region of Cu-Nb-3Gr exhibits greater structural stability, with a markedly lower number of splashed droplets and a relatively flat surface. The improved response originates from the effective synergy of the semi-continuous structure. On the one hand, the semi-continuous Gr-rich heat-conduction structure functions as a series of microscale heat-spreading fins, rapidly transporting transient heat from the arc root into deeper regions of the matrix. The resulting reduction in the peak temperature at the center of the molten bridge substantially decreases Cu vaporization, weakens the destructive vapor recoil force, and effectively suppresses the ejection of large Cu droplets. On the other hand, the high-melting-point Nb particles are dispersed relatively uniformly and remain solid at elevated temperatures. These particles are embedded in the liquid Cu, where they act as microscale flow barriers and increase the apparent viscosity of the solid–liquid mixture. The combined effects of thermal dissipation and physical support promote gradual extensional necking and fracture of the molten bridge, thereby effectively limiting splashing-induced erosion.

Notably, the Cu-Nb-5Gr simulation exhibits large-scale material detachment and asymmetric ejection of block-like fragments. The underlying mechanical origin of this behavior lies in severe phase agglomeration. Large Gr agglomerates, which introduce high effective electrical resistivity and interfacial thermal resistance, not only act as thermal barriers but also force the current to bypass the agglomerated regions. This diversion produces pronounced current crowding and severe local hot spots at the interfaces. Under the combined effects of high thermal stress and convective impact within the liquid metal, the relatively weak interfaces surrounding the agglomerates rapidly destabilize, causing macroscopic fragments together with liquid metal to be ejected from the contact surface.

Following molten bridge fracture and subsequent arc ignition, a molten pool forms on the material surface under the impingement of a high heat flux. The liquid metal within the molten pool undergoes complex heat- and mass-transfer dynamics jointly driven by the vapor recoil force, Marangoni convection, and electromagnetic forces. The top view of the molten pool in Figure 4d shows that the liquid Cu phase in yellow has a highly uneven distribution in the Cu-Nb-1Gr sample, with distinct local deep-melt regions. The isolated Nb phase in green and sparse Gr phase in purple are entrained by the vigorous convection of liquid Cu. This morphology indicates that, in the absence of effective heat-conduction pathways, the arc heat flux penetrates deeply into the material, inducing localized deep melting and pronounced fluid agitation. The high recoil pressure drives the liquid Cu radially outward, readily producing a highly undulating and rough surface after cooling and solidification. These dynamics agree well with the static experimental observations of preferential deep vaporization in the Cu-rich regions and the formation of peripheral melt zones.

Figure 4e presents a comparatively stable molten pool in Cu-Nb-3Gr. The high-melting-point Nb particles and the semi-continuous Gr-rich structure are relatively uniformly embedded within the shallow liquid Cu layer, and no evident macroscopic phase segregation is observed. This comparatively homogeneous fluid morphology indicates that the semi-continuous Gr-rich heat-conduction structure distributes the arc heat more uniformly across the surface, resulting in a wide and shallow molten pool. More importantly, the uniformly distributed solid Nb phase not only impedes the outward spreading of molten Cu but also exerts a hydrodynamic drag force on the vigorously circulating liquid Cu. Together with the reduced vapor recoil force, this drag effectively suppresses surface fluctuations driven by Marangoni convection. This dynamic flow-blocking mechanism maintains the morphological and dynamic stability of the molten pool and provides a plausible physical explanation for the smooth, gently undulating bright-white solidification striations observed at the erosion center in Figure 3e.

By contrast, Figure 4f reveals severe phase separation and fluid disorder. The agglomerated Gr phase in purple and Nb phase in green connect to form barriers that divide the liquid Cu matrix in yellow into fragmented islands. Because these large agglomerated regions resist melting and fail to move cooperatively with the surrounding fluid, they evolve into macroscopic defects within the molten pool. They not only impede the uniform dissipation of heat and induce abnormal boiling and splashing of liquid Cu within the narrow gaps but also become preferential sites for residual thermal-stress concentration and release during subsequent rapid solidification because of the pronounced differences in the coefficients of thermal expansion among the constituent phases. This dynamic evolution involving obstructed heat flow and thermal stress provides a direct mechanistic basis for the large thermal-stress cracks, deep erosion craters, and macroscopic material collapse at the phase interfaces observed experimentally in Figure 3i.

### 3.4. Wear Resistance of Cu-Nb-1Gr, Cu-Nb-3Gr, and Cu-Nb-5Gr Composites

**Figure 5:**
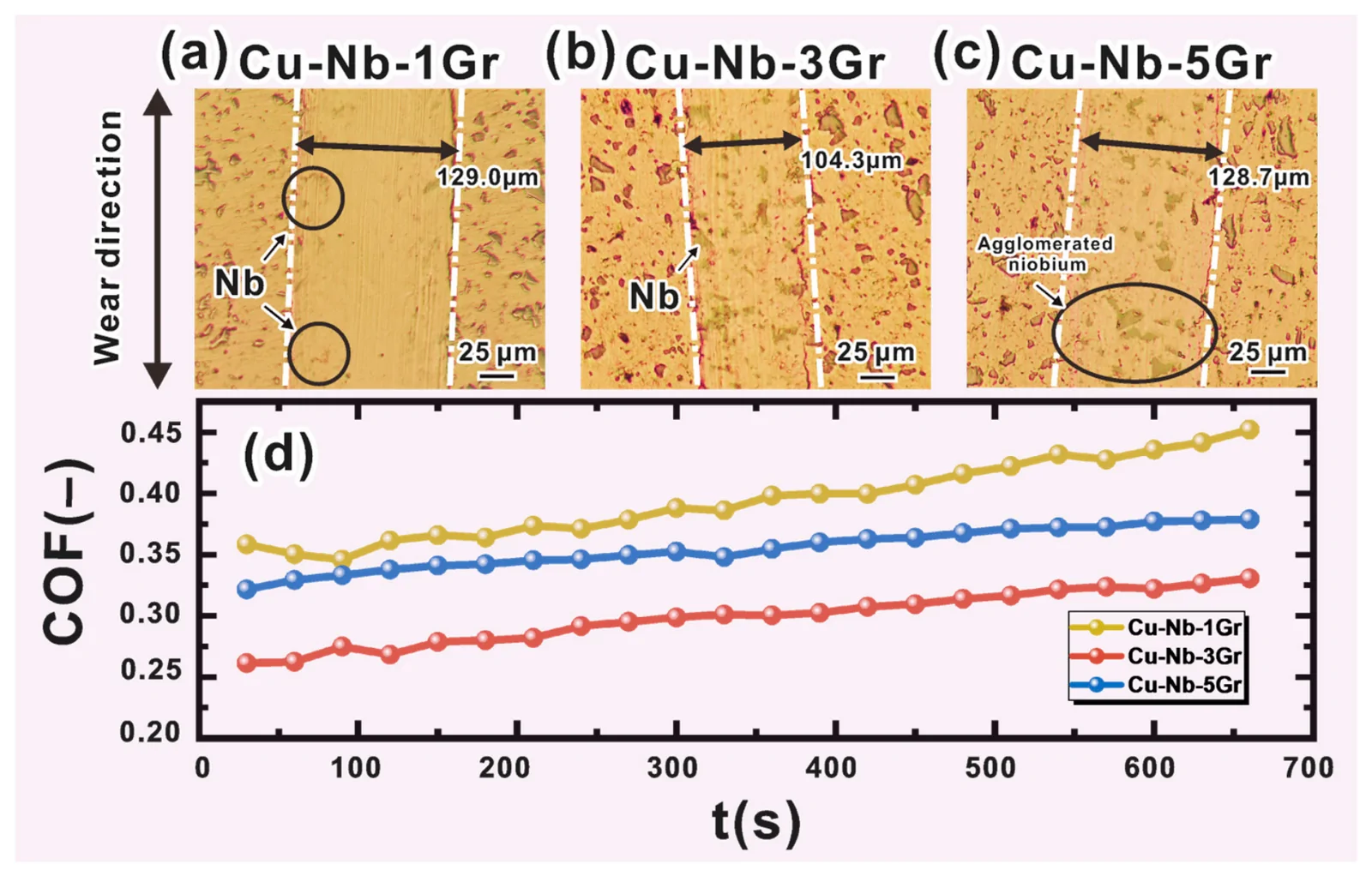
Friction and wear test results: (**a**–**c**) optical micrographs of the worn surfaces of Cu-Nb-1Gr, Cu-Nb-3Gr, and Cu-Nb-5Gr, respectively, and (**d**) the corresponding coefficient of friction (COF) evolution curves.

Taken together, the preceding static SEM characterization and multiphase-flow simulations demonstrate that the microscopic spatial topology of Gr plays a decisive role in governing the arc erosion resistance of the composites under extreme transient thermal shock. Under service conditions, however, the materials must withstand not only erosion by arc heat flux but also prolonged high-frequency sliding friction. Elucidating the potential influence of the evolution of this multiphase microarchitecture on dynamic friction behavior and wear resistance is therefore equally important. For this purpose, the samples were mounted on a tribometer, and dry-sliding tests were performed under a normal load of 2 N. Figure 5 presents the worn-surface morphologies and coefficient-of-friction (COF) curves. As shown in Figure 5a, after testing, the Nb phase within the central region of the wear track of Cu-Nb-1Gr is almost completely pulled out, numerous plowing grooves extend parallel to the sliding direction, and the Nb phase near the track edges also exhibits severe wear. Correspondingly, the COF curve (yellow solid line in Figure 5d) exhibits pronounced fluctuations and a rapid overall increase. Such behavior is primarily attributed to the low content of Gr as a solid lubricant, which cannot sustain a continuous lubricating transfer film at the sliding interface. Lubrication failure consequently exposes the soft Cu matrix to direct sliding contact with the counterface steel ball, facilitating local metal-to-metal adhesion and cold welding and thereby producing severe adhesive wear. Fragments of the damaged Cu matrix that are retained within the contact zone subsequently act as hard third-body abrasive particles. Driven by the counterface steel ball, these particles intensely plow the Cu matrix. As sliding proceeds, the grooves deepen and sliding resistance rises sharply, establishing a self-reinforcing deterioration process. The microstructure in Figure 2a further indicates that the fine and discretely distributed Nb particles cannot establish an effective Nb-rich load-bearing structure within the soft matrix. Under high normal and tangential stresses, these isolated hard particles not only fail to provide stable load-bearing support but are instead pressed directly into the Cu matrix or entrained in plastic flow with the matrix, further aggravating interfacial damage. The combined effects of these mechanisms result in the widest wear track and the poorest wear resistance for Cu-Nb-1Gr.

In contrast, Cu-Nb-3Gr (Figure 5b) exhibits markedly superior tribological performance. Its wear track remains relatively smooth, the plowing grooves are shallow, and the embedded Nb phase retains a distinct and nearly intact morphology. No evident pull-out damage is observed. Meanwhile, the COF curve of this sample is the smoothest and exhibits the smallest fluctuation amplitude, while stick–slip behavior produces neither spikes nor abrupt transitions. These characteristics indicate that the consumption and replenishment of the Gr lubricating film at the sliding interface attain a favorable dynamic equilibrium. As the surface material is progressively worn away, Gr exposed from the interior of the matrix is rapidly flattened and spread by alternating shear forces, continuously replenishing the lubricating film and preserving its integrity. In conjunction with Figure 2e, the more uniform Nb distribution and the moderate agglomeration of Nb particles promote the formation of a well-developed Nb-rich load-bearing structure within the matrix. Moreover, the semi-continuous Gr-rich structure preferentially surrounds the Nb phase and provides a bridging function, leading to the formation of a semi-continuous Gr-rich lubrication and heat-conduction structure coupled with an Nb-rich load-bearing structure. Within this optimized microarchitecture, the normal load is effectively borne by the Nb phase, while the surface solid-lubricant film is protected from excessive compression and disruption. The two phases therefore produce a pronounced synergistic effect on friction reduction and wear resistance, resulting in the lowest and most stable friction response throughout the entire sliding period.

The worn surface of Cu-Nb-5Gr (Figure 5c) exhibits differential wear. The Nb phase remains relatively intact within Nb agglomeration regions, whereas it is worn to varying degrees elsewhere. Although the COF curve of Cu-Nb-5Gr remains comparatively smooth, its absolute values are relatively high. In light of the severe Gr agglomeration evident in Figure 2h, this behavior reflects a steady-state wear regime with high sliding resistance. On the one hand, large quantities of detached Gr fragments and matrix debris form a thick third-body debris layer with sustained particle flow within the contact zone. This layer physically separates the two metallic surfaces and suppresses direct metal-to-metal adhesion and cold welding, thereby eliminating the pronounced sawtooth fluctuations in the COF curve. On the other hand, large Gr agglomerates provide almost no rigid support in the through-thickness direction. Consequently, the normal load from the steel ball induces rapid yielding and collapse of the material surface. The resulting sharp increase in the real contact area, together with the pronounced bulldozing effect generated as the counterface traverses the thick third-body debris layer, increases the macroscopic plowing resistance. Ultimately, the COF rises rather than decreases.

To elucidate the underlying dynamic mechanisms responsible for the aforementioned macroscopic friction and wear phenomena, and to verify from a theoretical perspective the decisive role of microstructural evolution in interfacial failure, FEM was further employed to reconstruct the dynamic wear processes of the three materials. The effects of Gr content on the friction mechanisms and deformation responses of the composites were systematically analyzed.

**Figure 6:**
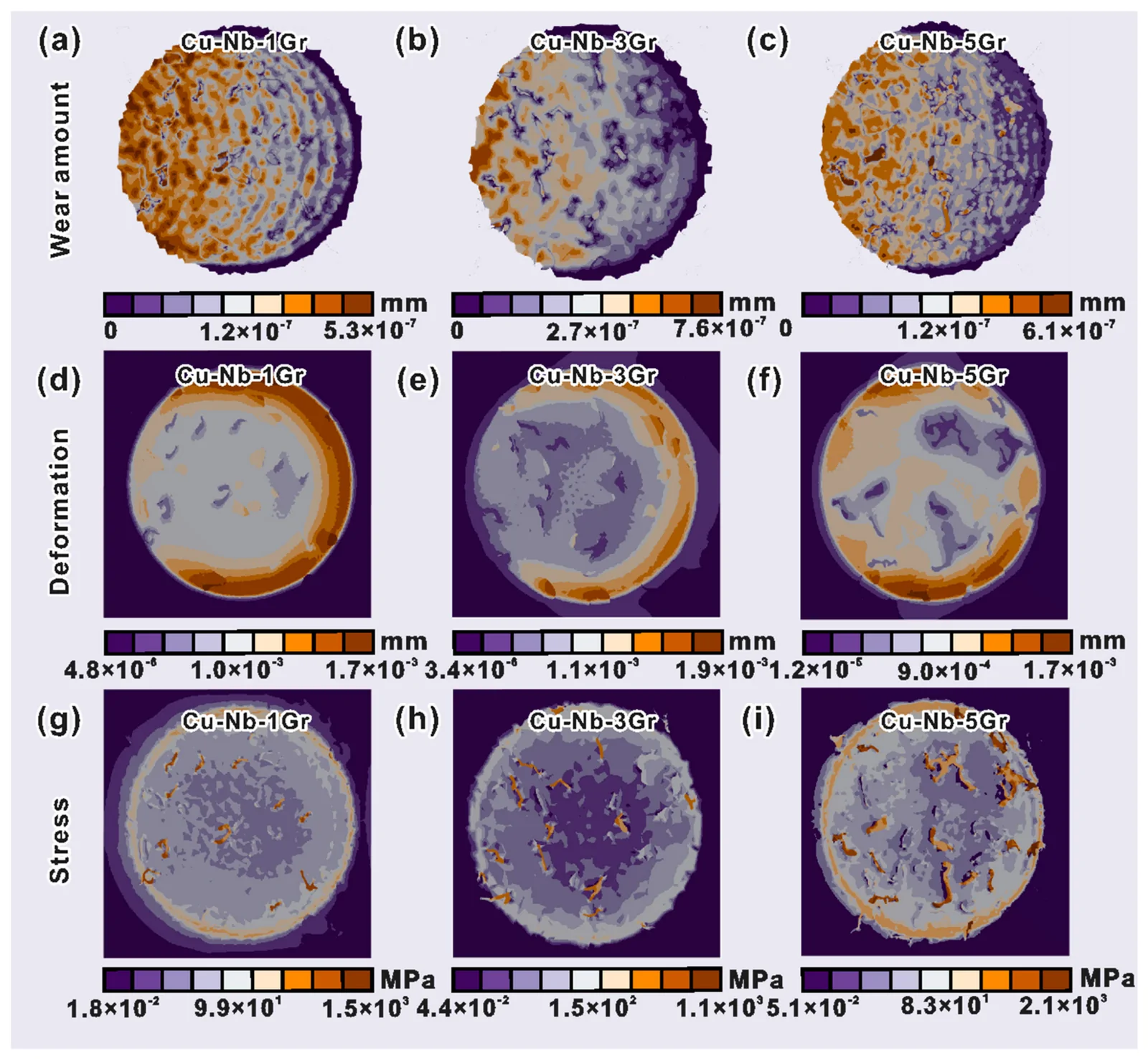
FEM simulation results for the materials after wear: (**a**–**c**) wear amount distributions, (**d**–**f**) total deformation distributions, and (**g**–**i**) equivalent stress distributions.

Figure 6 presents the mechanical and wear characteristics of the surfaces of the three composites after a single wear cycle. Overall, the simulated wear amounts agree closely with the experimental observations in Figure 5. As shown in Figure 6a, the surface of Cu-Nb-1Gr is almost entirely covered by light-brown regions denoting high wear amounts. The wear pattern exhibits an intermittent band-like distribution, with no evident undamaged regions. This result further confirms that the low Gr content cannot support the formation of a continuous protective lubricating film. Consequently, metal-to-metal cold-welded junctions are repeatedly ruptured during sliding, leading to severe adhesive material removal and relatively uniform but extensive material loss across the entire contact surface. By comparison, the surface of Cu-Nb-3Gr (Figure 6b) predominantly comprises light-colored or dark-blue regions of low wear, with only a few discrete orange peaks of locally high wear. This simulation result is broadly consistent with the mechanism inferred from Figure 5, in which the uniform spreading of the Gr lubricating film effectively protects the matrix. In contrast, the mottled high-wear regions on the surface of Cu-Nb-5Gr (Figure 6c) directly reflect the brittle spallation of macroscopic Gr agglomerates arising from insufficient interfacial bonding. The detached hard fragments are retained within the contact zone, where they induce severe three-body abrasive wear. Therefore, although the high Gr content provides an overall lubricating environment superior to that of Cu-Nb-1Gr, the local spallation of the severely agglomerated phase causes the overall wear amount to substantially exceed the corresponding values for the other two samples.

The total deformation distributions of the three materials are presented in Figure 6d–f. Cu-Nb-1Gr exhibits the largest overall deformation. The formation of a broad and well-defined brown annular region of high deformation is evident along the edge, while the surface deformation distribution remains nonuniform and only the matrix adjacent to the Gr phase exhibits relatively low deformation. These features indicate that the incorporation of Gr mitigates plastic deformation. However, the low Gr content and small Nb particle size preclude effective load-bearing support, resulting in substantial overall deformation. Cu-Nb-3Gr exhibits a marked improvement (Figure 6e). Most of the surface comprises light-colored or dark-blue regions of low deformation, while band-like regions of relatively high deformation are confined to the edge. Particularly in the central region, the semi-continuous distribution of Gr-rich regions results in the formation of a dark-blue, island-like network of low deformation. This superior deformation resistance is attributed to the synergistic action of the semi-continuous Gr-rich structure, which promotes the formation of an effective Nb-rich load-bearing structure by Nb particles of an appropriate size. Within this semi-continuous structure, the semi-continuous Gr-rich structure efficiently absorbs and accommodates most of the friction-induced shear strain, while the Nb-rich load-bearing structure constrains macroscopic yielding and plastic flow of the Cu matrix. A favorable strength–ductility balance is thereby achieved. By contrast, severe phase agglomeration in Cu-Nb-5Gr disrupts this synergistic mechanism. As shown in Figure 6f, only the Gr agglomeration regions with a high elastic modulus retain relatively low deformation. The Cu matrix surrounding these agglomerates, together with the central matrix region, is subjected to localized compression, producing light-brown or dark-brown collapse zones with severe deformation.

On the basis of the preceding deformation analysis, the equivalent stress distribution at the sliding interface is further examined in detail. The results show that Cu-Nb-1Gr exhibits a generally high stress level, with the annular high-stress region along the edge being particularly prominent. Numerous discrete point-like high-stress regions are distributed across the surface and spatially coincide with the Gr phase. Because the elastic modulus of Gr far exceeds those of Nb and Cu, the high-modulus Gr phase acts as a local stress-concentration hotspot under external loading. However, its sparse and discontinuous distribution cannot establish an effective stress-transfer network. By comparison, the Gr phase in Cu-Nb-3Gr forms a semi-continuous high-stress band distributed along the wear track, and the stress gradient from the Gr band toward the adjacent Cu matrix decays relatively smoothly. This behavior is attributed to the stress-transfer pathway provided by the semi-continuous Gr-rich structure. The load rapidly spreads laterally through the semi-continuous Gr-rich structure. Consequently, the originally isolated high-stress points are connected and redistributed into a uniform stress field. The semi-continuous Gr-rich structure bears a stable, spatially distributed load and continuously transfers the load to the surrounding Cu matrix through effective interfacial shear coupling, thereby enabling cooperative load bearing. The gradual lightening of the Cu-matrix color from the center toward the periphery in the contour map provides a direct physical representation of the efficient and uniform transfer of stress into the surrounding matrix. In contrast, large high-stress islands appear on the surface of Cu-Nb-5Gr. Their spatial contours closely coincide with those of the Gr agglomerates, and deep-blue low-stress regions indicative of stress shielding surround the high-stress islands. This defect originates from the severe elastic–modulus mismatch. As highly rigid structures, the Gr agglomerates cannot deform compatibly with the surrounding soft matrix. Under external loading, smooth load transfer is obstructed, causing stress to become trapped at the weak Gr/Cu interfaces and resulting in the formation of extreme interfacial stress concentrations. Such microscopic agglomeration disrupts the continuous stress field, producing regions of severe interfacial stress concentration alongside low-stress matrix regions that contribute little to load bearing. The interruption of the load-bearing pathways and the stress concentrations at the interfaces directly drive the brittle disintegration of the agglomerates and the consequent deterioration in the wear rate of Cu-Nb-5Gr.

**Figure 7:**
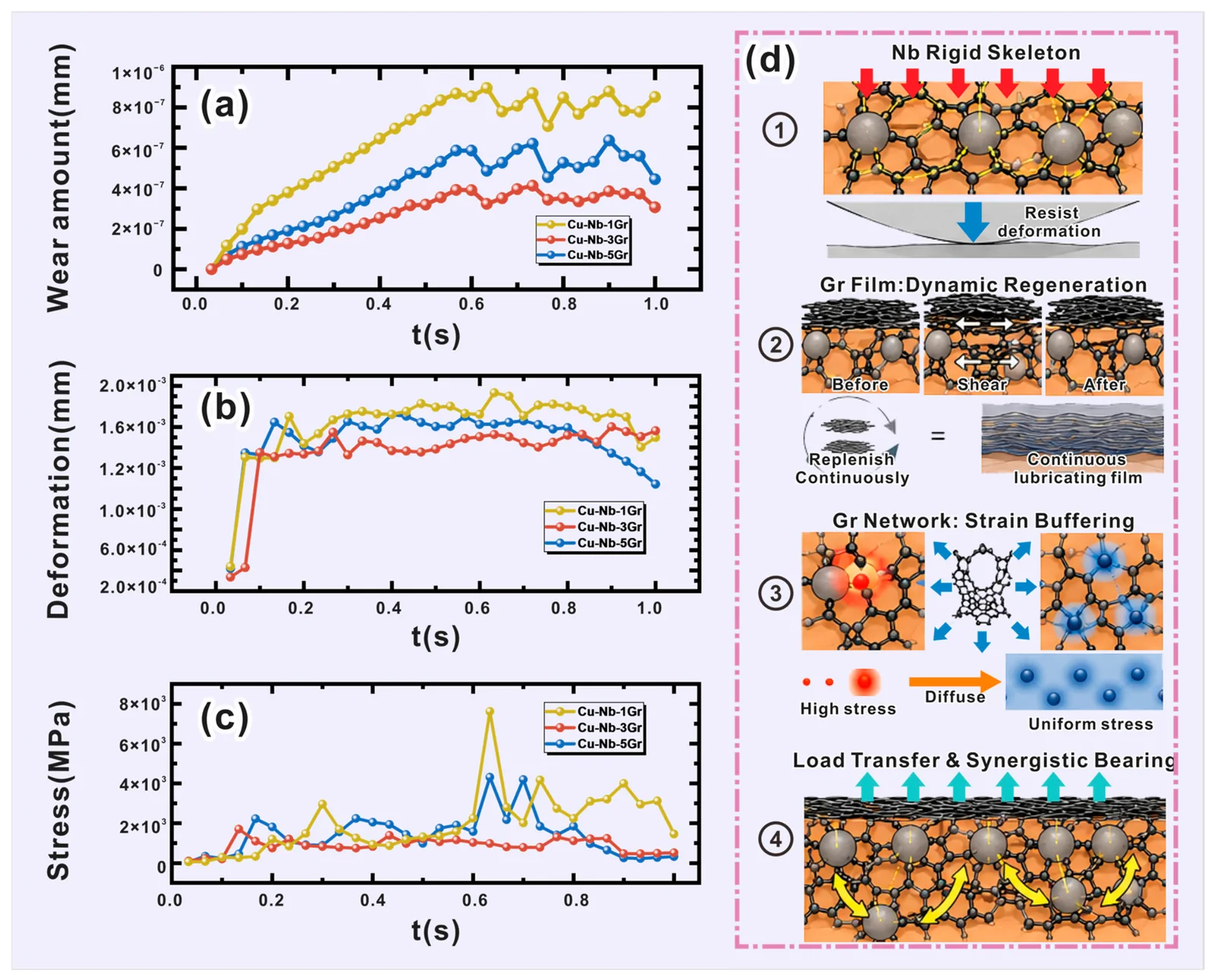
Dynamic response curves during sliding: (**a**) temporal evolution of wear amount, (**b**) temporal evolution of total deformation, (**c**) temporal evolution of average equivalent stress, and (**d**) schematic illustration of the mechanism by which Gr enhances the wear resistance of the material.

Building on the preceding static analysis of surface damage morphology and the spatial distributions of mechanical responses after a single wear cycle, the temporal evolution curves of wear amount, total deformation, and average equivalent stress for the three composites were quantitatively analyzed, as shown in Figure 7, to further elucidate the evolution of frictional dynamics and the dynamic failure mechanisms of the materials. Figure 7a presents the variations in wear amount with time for the three samples. During the initial running-in stage, the wear amounts of all three samples increase steeply, and the initial wear rates follow the sequence Cu-Nb-1Gr > Cu-Nb-5Gr > Cu-Nb-3Gr. The sequence provides preliminary evidence for the critical role of Gr as a solid lubricant in suppressing initial wear. As sliding continues beyond approximately 0.58 s, the three curves begin to diverge. The wear curve of Cu-Nb-1Gr exhibits large-amplitude, irregular oscillations, a characteristic signature of interfacial instability during adhesive wear, particularly repeated stick-slip behavior. By contrast, the wear curve of Cu-Nb-3Gr remains nearly horizontal after reaching the steady state, indicating that an optimal dynamic balance develops between the depletion and replenishment of the Gr lubricating film at the sliding interface. The near-horizontal response agrees closely with the smooth worn surface observed in Figure 5. Although the wear curve of Cu-Nb-5Gr exhibits comparatively regular oscillations, the peak amplitudes progressively increase and the oscillation periods lengthen with time. These progressive changes indicate that the material has entered a sustained regime of three-body abrasive wear. Severe phase agglomeration compromises structural integrity, causing large Gr agglomerates and some unsupported Nb particles to be continuously detached from the matrix and converted into loose hard third-body particles. These detached particles form a dynamically balanced particle flow at the contact interface, which subjects the matrix to continuous and uniform micro-cutting while physically separating the metallic surfaces. Abrupt changes in frictional resistance are thereby suppressed, consistent with the relatively smooth COF curve of this sample in Figure 5d. Nevertheless, this friction regime depends on extensive spallation of the material itself and therefore produces continuous matrix loss. Meanwhile, the repeated rolling and cutting actions of the third-body particles further intensify surface fatigue damage, ultimately causing a substantial deterioration in the overall wear resistance of the composite.

Figure 7b reveals the evolution of macroscopic deformation. During the initial stage of wear, at approximately 0.1 s, sudden load application drives the deformation of all three samples rapidly to peak values. Cu-Nb-1Gr and Cu-Nb-5Gr exhibit comparable peaks of approximately 1.7 × 10^−3^ mm, slightly higher than the value of 1.5 × 10^−3^ mm for Cu-Nb-3Gr. After deformation reaches the steady state, the total deformation follows the sequence Cu-Nb-1Gr > Cu-Nb-5Gr > Cu-Nb-3Gr. Owing to insufficient load-bearing support in Cu-Nb-1Gr, extensive plastic flow develops in the Cu matrix, producing the largest total deformation. Cu-Nb-3Gr benefits from the formation of a semi-continuous Gr-rich structure coupled with an Nb-rich load-bearing structure. These two structural components reduce interfacial shear stress and effectively constrain matrix yielding, thereby maintaining the total deformation at the lowest level. The deformation of Cu-Nb-5Gr lies between those of the other two samples. Notably, the total deformation curve of this sample exhibits a pronounced decline during the final stage of wear. Together with the continuously increasing wear amount in Figure 7a, this late-stage decline indicates that, as particle spallation intensifies, a substantial fraction of the loose hard debris is repeatedly pressed back into the softer Cu surface and mechanically interlocked with the near-surface matrix under repeated rolling at the sliding interface. Repeated re-embedding leads to the formation of a dense, hard-particle-rich mechanically mixed layer. The resulting rigid load-bearing bed is composed of high-modulus spalled fragments and carries most of the normal load, thereby markedly suppressing macroscopic plastic deformation of the underlying Cu matrix during the later stage.

Figure 7c further characterizes the evolution of internal stress under frictional shear. The average equivalent stress curve of Cu-Nb-1Gr exhibits multiple extremely high stress peaks that far exceed those of the other two samples. The experimental results in Figure 5 indicate that, in the absence of lubricating protection, cold-welded junctions frequently develop between the Cu matrix and the steel ball. Rupture of each cold-welded junction requires a very high shear force to overcome metallic bonding, directly producing the pronounced stress excursions in the curve. By contrast, Cu-Nb-3Gr exhibits the smoothest average equivalent stress curve, without evident stress peaks. This response is attributed to the spreading of extruded Gr across the sliding surface and the resulting formation of a continuous solid-lubricant film. The film transforms the contact mode from direct contact between Cu and the steel ball to contact between the steel ball and the Gr-rich film, accompanied by interlayer sliding within Gr. Accordingly, the dominant resistance to sliding shifts from the high shear resistance of metallic cold-welded junctions to the weak interlayer resistance governed by van der Waals interactions between Gr layers. Rapid unloading of the interfacial stress is consequently promoted, and the stress state becomes uniform and stable, clearly demonstrating the superior self-repair capability of the lubricating film. The average equivalent stress curve of Cu-Nb-5Gr also exhibits fluctuations, although its peak values remain markedly lower than those of Cu-Nb-1Gr. The lower peaks arise because the dominant frictional resistance shifts from metal-to-metal adhesion and tearing to rolling and displacement within the third-body particle flow. Overcoming the internal friction and rolling resistance among these loose abrasive particles consumes more energy than ideal interlayer sliding of Gr but far less energy than rupturing metallic cold-welded junctions. Accordingly, the equivalent stress level of Cu-Nb-5Gr falls between those of Cu-Nb-1Gr and Cu-Nb-3Gr.

### 3.5. Compression Resistance of Cu-Nb-1Gr, Cu-Nb-3Gr, and Cu-Nb-5Gr Composites

**Figure 8:**
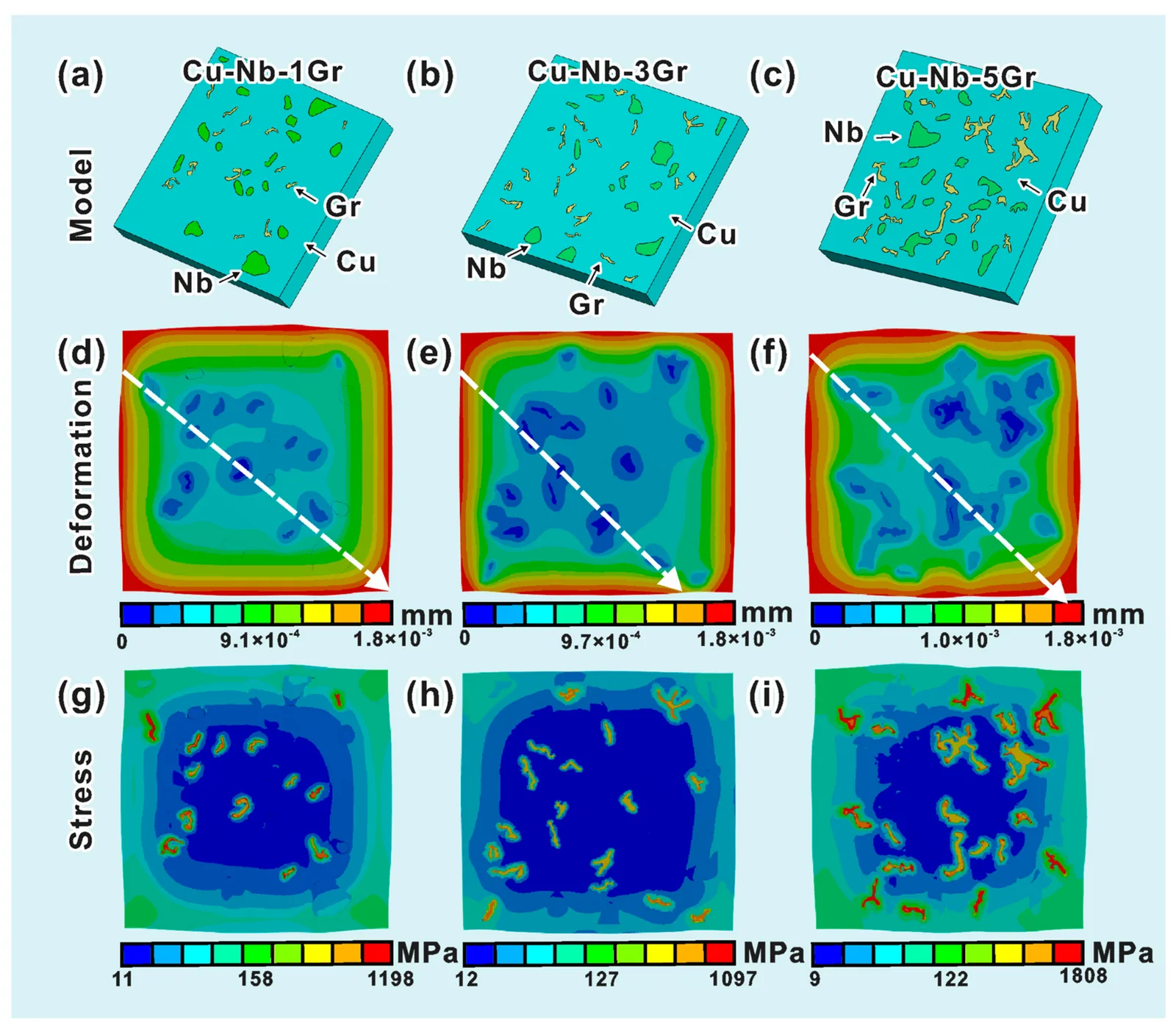
FEM simulations of mechanical behavior and stress–strain partitioning under compressive loading: (**a**–**c**) three-dimensional structural models reconstructed from the actual microstructures, (**d**–**f**) simulated total deformation contour maps, and (**g**–**i**) equivalent stress contour maps.

In addition to the aforementioned sliding wear, materials in practical service are frequently subjected to high-intensity compressive impact loads, which pose severe challenges to macroscopic structural stability and fatigue life. Elucidating how the microstructure, particularly the spatial distribution of Gr, governs the macroscopic compression resistance of the composites is therefore essential. Based on the actual microscopic structural features observed in Figure 2, three-dimensional structural models of the three composites were reconstructed using SolidWorks (Figure 8a–c). The mechanical responses and failure mechanisms under hydrostatic compressive loading were subsequently investigated in detail using FEM.

Figure 8d–f present the total deformation contour maps of the three samples. In Cu-Nb-1Gr, extensive and relatively uniform subsidence occurs in the central region, as indicated by the light blue-green color. The formation of a continuous annular deep-red region of high deformation is observed along the edge. These features indicate that the low content and discrete distribution of the reinforcing phases cannot establish an effective load-bearing structure, causing macroscopic plastic yielding throughout the Cu matrix under compression. In Cu-Nb-3Gr, the high-deformation regions are markedly suppressed, and the color transition across the entire deformation field remains extremely gradual. No evident localized deformation anomaly is observed. In particular, the blue low-deformation regions interconnect and coalesce into an extensive continuous domain at the sample center, with their spatial distribution closely corresponding to the semi-continuous Gr-rich structure. This result provides strong evidence that the semi-continuous Gr-rich structure cooperates with Nb particles of an appropriate size to promote the formation of an effective Nb-rich load-bearing structure within the matrix. The Nb-rich load-bearing structure supports and redistributes a substantial proportion of the macroscopic compressive load and enables strain delocalization and compatible deformation between the matrix and reinforcing phases, thereby markedly enhancing the overall compressive stiffness of the composite. By contrast, Cu-Nb-5Gr exhibits the most severe macroscopic collapse along the edge, together with a highly nonuniform deformation field. Only a few isolated low-deformation regions appear near the Gr agglomerates, whereas severe dark-colored high-deformation regions develop in the surrounding Cu matrix. The underlying mechanical origin of this behavior lies in the pronounced nonuniformity of strain partitioning. Because the stiff Gr agglomerates cannot deform compatibly with the matrix, compressive deformation is forced to localize within the constrained Cu regions between adjacent agglomerates. The resulting extreme local compressive strains induce plastic distortion and material collapse near the interfaces.

After the decisive influence of Gr distribution on macroscopic compressive deformation was established, the equivalent stress distributions within the materials were further analyzed in detail, as shown in Figure 8g–i. In Cu-Nb-1Gr, most of the matrix appears dark blue, corresponding to low-to-moderate stress levels, while only a few discrete orange-red high-stress points are scattered at the tips of isolated Gr platelets. During load transfer from the Cu matrix to the high-modulus Gr platelets, characteristic local stress perturbations develop at the phase interfaces, accompanied by stress concentrations at the platelet tips. For Cu-Nb-3Gr, the deep-blue low-stress region covers the largest surface area, and almost no prominent orange-red high-stress hotspots are present. This result demonstrates that the semi-continuous Gr-rich structure constitutes an efficient stress-transfer pathway within the material. The external load is rapidly redistributed through this semi-continuous structure to the Nb-rich load-bearing structure and Cu matrix, fully exploiting the advantages of Nb-Gr multiphase load sharing. Such pronounced stress homogenization effectively prevents extreme local stress accumulation and provides greater tolerance to compressive damage. In sharp contrast, the deep-blue low-stress region of Cu-Nb-5Gr contracts substantially, while numerous bright-red high-stress islands appear across the surface. The spatial contours of these high-stress regions closely coincide with those of the Gr agglomerates. The fundamental mechanical origin of this behavior lies in the extreme elastic–modulus mismatch. The large rigid Gr agglomerates act as structural inclusions within the material. Under compression, the load-transfer paths are interrupted by these structural obstacles, and stress is consequently localized at the weak Gr/Cu interfaces. Extreme stress concentrations that far exceed the yield limit of the matrix are therefore generated at the interfaces. Such agglomeration-induced interfacial overstress triggers microcrack initiation and propagation during the initial loading stage and directly promotes premature macroscopic brittle fracture under compressive-impact service conditions. Therefore, Gr agglomeration must be strictly controlled during the design and fabrication of the composites.

**Figure 9:**
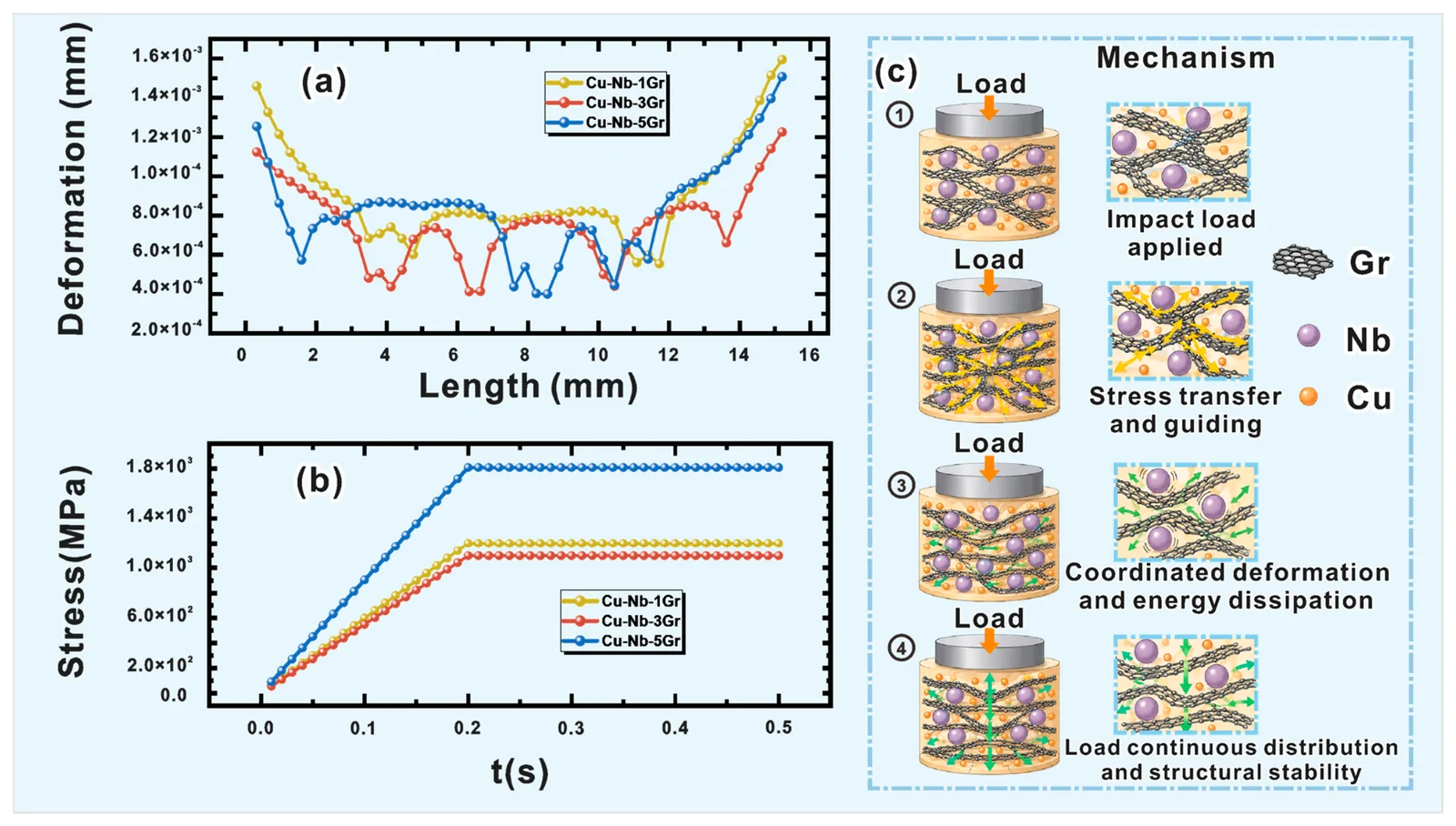
Dynamic response during compressive impact: (**a**) total deformation distribution along the diagonal extraction path, (**b**) temporal evolution of the average equivalent stress, and (**c**) schematic illustration of the synergistic deformation and stress-transfer mechanisms of the multiphase system.

To convert the spatial morphological features in the contour maps into precise quantitative descriptions of mechanical evolution and to comprehensively elucidate the strain-partitioning and stress-dissipation mechanisms of the three composites during compressive impact, the total deformation profiles along the diagonal of the contact surface (the white dashed line in Figure 8) were further extracted and analyzed (Figure 9a), together with the evolution curves of the surface-averaged equivalent stress (Figure 9b). The extraction path was selected to intersect as many regions of the matrix and reinforcing phases as possible and was fixed at identical coordinates in the three-dimensional models of the three composites to ensure rigorous comparison.

The deformation profiles along the diagonal (Figure 9a) of all three materials exhibit a characteristic broad U-shaped macroscopic distribution, with the maximum deformation at both ends and the minimum deformation in the middle. From the perspective of solid mechanics, the specimen edges undergo uniaxial compression with minimal lateral constraint, permitting relatively free outward plastic flow and consequently producing the maximum deformation. By contrast, the central region experiences a complex triaxial stress state and strong geometric constraint from the surrounding material. The surrounding material therefore markedly restricts plastic flow, resulting in the minimum deformation. After the effect of macroscopic geometric constraint is excluded, pronounced differences in microscopic deformation become evident. The deformation profile of Cu-Nb-1Gr exhibits a broad U-shaped contour with minimal local fluctuations but a relatively high overall deformation. This behavior indicates that the low content and discrete distribution of the reinforcing phases, including fine Nb particles and sparse Gr domains, cannot establish an effective structural support network. Under compressive stress, widespread and relatively uniform plastic flow develops in the Cu matrix. The hard phases provide little effective load-bearing support and consequently produce no substantial improvement in resistance to macroscopic compressive yielding. By comparison, the deformation profile of Cu-Nb-3Gr exhibits regular wave-like oscillations, while the overall macroscopic deformation is effectively suppressed. This superior response arises from the formation of an optimal internal microarchitecture. The semi-continuous Gr-rich structure and Nb particles of moderate size jointly establish a semi-continuous load-bearing structure within the matrix. When the extraction path traverses these high-stiffness reinforcing phases, the local deformation decreases sharply, giving rise to deep troughs in the curve. When the path traverses Cu-matrix regions adjacent to the Nb-rich load-bearing structure, moderate compatible deformation occurs, producing peaks. These regular and gradual microscopic strain fluctuations provide compelling evidence that the matrix and reinforcing phases undergo effective compatible deformation. This response not only suppresses macroscopic plastic collapse but also promotes a smooth transition of internal strain, thereby mitigating localized damage. In contrast, the deformation profile of Cu-Nb-5Gr exhibits irregular oscillations, with alternating deep troughs and high peaks. This pattern indicates highly nonuniform strain partitioning. The high-modulus Gr agglomerates undergo only limited deformation, corresponding to the troughs. By severely constraining the available deformation space, these agglomerates force the surrounding Cu matrix to accommodate intense local compressive strain and undergo severe collapse, thereby generating the peaks. The pronounced deformation mismatch between adjacent microregions directly drives shear-induced tearing within the material.

The evolution curves of the surface-averaged equivalent stress are presented in Figure 9b. The compression simulation was designed to compare the transient stress and deformation responses of the three composites under short-duration loading rather than to reproduce long-term strain-hardening or dynamic-recovery processes. All three curves increase rapidly from 0 to 0.2 s as the applied pressure rises linearly from 0 to 27.6 MPa. The plateau-like response during the interval from 0.2 to 0.3 s corresponds to the prescribed load-holding stage, during which the applied pressure remains constant at 27.6 MPa. Accordingly, the plateau represents a numerically stable stress response during transient load holding and is not interpreted as evidence of a metallurgical balance between strain hardening and dynamic recovery. Figure 9b is therefore used only to compare the relative mechanical responses of the three microstructures under identical transient-impact conditions. During the load-holding stage, Cu-Nb-3Gr exhibits the lowest surface-averaged equivalent stress, approximately 1097 MPa. This favorable response is attributed to the optimal microstructural configuration. The semi-continuous Gr-rich structure and Nb particles cooperatively establish efficient stress-transfer pathways throughout the matrix. Through these pathways, the applied compressive load is smoothly redistributed across the Cu matrix and reinforcing phases, enabling global cooperative load sharing throughout the multiphase architecture. This efficient stress-transfer mechanism alleviates localized stress accumulation and allows the material to retain greater structural integrity under transient loading. Cu-Nb-1Gr exhibits an intermediate surface-averaged equivalent stress during the load-holding stage. Because the reinforcing phases occur at a low content and with a discrete distribution, an effective stress-transfer pathway cannot develop. Under transient compression, these spatially discrete hard particles impede local deformation and produce moderate stress concentrations along the particle edges.

By contrast, Cu-Nb-5Gr exhibits the highest surface-averaged equivalent stress during the load-holding stage. This exceptionally high stress level fundamentally originates from the severe macroscopic agglomeration resulting from excessive Gr. Under macroscopic compression, a pronounced elastic–modulus mismatch develops between the agglomerates and matrix. The high-modulus Gr agglomerates cannot participate effectively in compatible deformation. Consequently, load transfer is severely impeded, and extreme local stress concentrations are generated at the weak Gr/Cu interfaces. This high numerical stress response indicates impaired interfacial load transfer and pronounced stress concentration under the prescribed transient loading, rather than a steady-state flow-stress plateau governed by metallurgical recovery. These stress concentrations may increase susceptibility to microcrack initiation and propagation under compressive-impact service conditions, potentially leading to premature macroscopic brittle failure.

## 4. Conclusions

Cu-Nb-Gr ternary composites were fabricated via high-energy ball milling followed by FHPS. Experimental characterization was integrated with CFD and FEM simulations to elucidate the mechanisms governing the effects of Gr on dynamic arc erosion, compressive deformation, and dry sliding wear behavior. The main conclusions are as follows:

(1) The volume fraction of Gr directly affects the microstructure and surface-damage morphologies of the Cu-Nb-Gr composites. A Gr content of 3 vol.% enables the formation of a semi-continuous Gr-rich structure coupled with an Nb-rich load-bearing structure. This semi-continuous structure promotes heat dissipation and stress homogenization across the contact surface; reduces the formation of surface defects such as erosion pits, thermal-stress cracks, and plowing grooves; and thereby effectively mitigates erosion and wear damage. By contrast, an excessive Gr content of 5 vol.% causes severe phase agglomeration, leading to increased local thermal resistance and extreme stress concentrations and consequently intensifying structural degradation.
(2) The semi-continuous structure plays a critical role in regulating arc erosion and molten pool evolution. The semi-continuous Gr-rich heat-conduction structure establishes stable and efficient in-plane heat-conduction pathways, thereby lowering the peak temperature of the molten pool and weakening the metal vapor recoil force. Consequently, the boiling and spattering of liquid Cu are effectively suppressed, while the controlled fracture of the molten bridge is accelerated. Meanwhile, the uniform distribution of the high-melting-point Nb phase impedes intense convection and outward spreading of the liquid metal. The synergistic action of these two constituents maintains both the static and dynamic stability of the molten pool surface, thereby significantly enhancing the arc erosion resistance of the material.
(3) The synergistic effect of the semi-continuous structure simultaneously enhances the compressive load-bearing capability, friction-reduction performance, and wear resistance of the Cu-Nb-3Gr composite. During mechanical loading and sliding friction, Gr at the contact interface undergoes dynamic spreading, enabling the formation of a solid lubricating film and reducing the COF and interfacial shear stress. The Nb-rich load-bearing structure provides stable support under normal loading. The strong synergy within this semi-continuous structure alleviates the elastic–modulus mismatch and stress concentrations at the interfaces between the reinforcing phases and Cu matrix. Interfacial debonding, brittle spalling, and macroscopic collapse of the matrix are consequently suppressed, effectively reducing the average deformation and wear amount.

## Figures and Tables

**Table 1:** Species properties, boundary conditions, and model parameters.

Parameter	Cu	Nb	Gr	Cu (Vapor)
Atomic weight/(g·mol^−1^)	63.55	92.91	12.01	63.55
Melting point/(K)	1358	2750	4000	—
Density/(g·cm^−3^)	8.96	8.57	2.26	5.6 × 1^−3^
Specific heat/(J·kg^−1^·K^−1^)	385	265	710	327
Viscosity/(kg·m^−1^·s^−1^)	3.2 × 10^−3^	5.0 × 10^−3^	—	4.0 × 10^−5^
Heat conductivity/(W·m^−1^·K^−1^)	4.00 × 10^2^	0.54 × 10^2^	53.0 × 10^2^	0.01
Thermal expansion coefficient/(K^−1^)	16.5 × 10^−6^	7.3 × 10^−6^	4.0 × 10^−6^	3.5 × 10^−4^
Minimum temperature/(K)	300			
Maximum temperature/(K)	2300			
Temperature/Sliding cycles	50 1 × 10^−4^ Adiabatic and non-slip			
Grid size/(mm)				
Walls/(—)				

**Table 2:** Electrical conductivity, hardness, and density data for the Cu-Nb-1Gr, Cu-Nb-3Gr, and Cu-Nb-5Gr samples.

Materials	Electrical Conductivity (%IACS)	Hardness (HV)	Density (g·cm^−3)^
Cu-Nb-1Gr	78.89	73.98	8.75
Cu-Nb-3Gr	73.45	77.16	8.58
Cu-Nb-5Gr	67.11	76.30	8.45

## Data Availability

The original contributions presented in this study are included in the article. Further inquiries can be directed to the corresponding authors.

## References

[1] Lee R.-M., Chen J.-C., Liu J.-H. (2025). Advanced analysis of main bearing failure modes and design optimization in MW-sized wind turbines: A multi-physics coupling theoretical framework. Eng. Fail. Anal..

[2] Wang Y., Li J., Guo S., Sun M., Zhao H., Wu Y., Zhao L., Wang Z. (2024). Parameter sensitivity analysis of a multi-physics coupling aging model of lithium-ion batteries. Electrochim. Acta.

[3] Xu H., Huang X., Wang Y., Lou Y., Liu Z., Li B. (2025). Multi-physics coupling model-based numerical analysis of the effect of electric current path on large cross-section electroslag fusion welding. Ironmak. Steelmak..

[4] Sun R., Jeong H., Zhao J., Gou Y., Sauret E., Li Z., Gu Y. (2024). A physics-informed neural network framework for multi-physics coupling microfluidic problems. Comput. Fluids.

[5] Zhao X., Ren X., Chen Z., Ge C. (2024). Enhancement of mechanical and electrical properties of copper matrix composites by different types of carbon nanotubes. Mater. Charact..

[6] Li T., Wang Y., Yang M., Hou H., Wu S. (2021). High strength and conductivity copper matrix composites reinforced by in-situ graphene through severe plastic deformation processes. J. Alloys Compd..

[7] Xue Z.W., Liu Z., Wang L.D., Fei W.D. (2010). Thermal properties of new copper matrix composite reinforced by β-eucryptite particulates. Mater. Sci. Technol..

[8] Jahani A., Jamshidi Aval H., Rajabi M., Jamaati R. (2023). Effects of Ti2SnC MAX Phase on Microstructure, Mechanical, Electrical, and Wear Properties of Stir-Extruded Copper Matrix Composite. Adv. Eng. Mater..

[9] Xiao Y., Shen L., Zhao H., Cheng Y., Du J., Shen M. (2024). Tribological properties of copper-matrix composites against laser-cladded Co06 coating on 30CrMoSiVA steel at elevated temperatures. Tribol. Int..

[10] Zeng L., Liang Y., Chen P. (2024). In-situ grown continuous graphene network enhances the electrical conductivity and tribological properties of copper matrix composites. Front. Mater. Sci..

[11] Yang Y., Guo X., Song K., Long F., Wang X., Li S., Li Z. (2022). Electrical wear performance of copper matrix composites reinforced with hybrid CNTs and TiB_2_ particles. Ind. Lubr. Tribol..

[12] Yang T., Chen W., Yan F., Lv H., Fu Y.Q. (2021). Effect of reduced graphene oxides decorated by Ag and Ce on mechanical properties and electrical conductivity of copper matrix composites. Vacuum.

[13] Dong B., Guo X., Tong P., Xie L., Liu K., Xiong T., Zhu X., Lin J., Song W., Sun Y. (2024). A zero-thermal-expansion composite with enhanced thermal and electrical conductivities resulting from 3D interpenetrating copper network. J. Alloys Compd..

[14] Tong Y., Wang L., Wang B., Hu Y., Cai Z., Ren J., Liu J., Li S. (2023). Microstructure and mechanical behavior of carbon fiber reinforced carbon, silicon carbide, and copper alloy hybrid composite fabricated by Cu-Si alloy melt infiltration. Adv. Compos. Hybrid Mater..

[15] Yaman K. (2022). Fractal characterization of electrical conductivity and mechanical properties of copper particulate polyester matrix composites using image processing. Polym. Bull..

[16] Koltsova T.S., Bobrynina E.V., Larionova T.V., Salynova M.A., Tolochko O.V. (2022). Structure and properties of copper-based composite with different types of carbon nanostructures. Diam. Relat. Mater..

[17] Das R., Katiyar N.K., Khan A., Owuor P.S., Ike S., Sarkar S., Mishra V., Sarkar S., Tiwary C.S. (2025). Enhanced mechanical, tribological, and thermal properties of copper-graphene composites using additive manufacturing. J. Manuf. Process..

[18] Bang J., Kim Y., Lee E. (2025). High-temperature mechanical and wear behavior of hypoeutectic Al-Si-(Cu)-Mg alloys with hardening mechanisms dictated by varying Cu:Mg ratios. Appl. Sci..

[19] Guo X., Liu T., Song Y., Li R., Wei P., Liao Z., Wu Z., Gao D., Fu Q., Wang G. (2025). Selective CO electroreduction to multicarbon oxygenates over atomically dispersed Cu-Ag sites in alkaline membrane electrode assembly electrolyzer. Angew. Chem. Int. Ed..

[20] Wang P., Wu Y., Hou H., Hao Z., Zhang S., Liang M., Li J. (2026). Hot deformation characteristics and hot processing map analysis of Cu-Nb composite wires reinforced by niobium strip. J. Mater. Eng. Perform..

[21] Artzt K., Siggel M., Kleinert J., Riccius J., Requena G., Haubrich J. (2020). Pyrometric-Based Melt Pool Monitoring Study of CuCr1Zr Processed Using L-PBF. Materials.

[22] Derby B.K., Gomez-Hurtado L.R., Copeland G., Hattar K., Briggs S. (2023). Deposition-Controlled Phase Separation in CuNb Metallic Alloys. Thin Solid Films.

[23] Wu Y., Hao Z., Hou H., Zhang S., Li J., Wang P. (2026). Tunable microstructure and properties of fiber-reinforced Cu-Nb composite through annealing process. J. Mater. Eng. Perform..

[24] Ding C., Li H., Xu J., Shan D., Guo B. (2025). Deformation behavior and strengthening mechanism of Cu/Nb nanolayered composites: Molecular dynamics simulation and experimental verification. J. Mater. Sci..

[25] Cheng J.Y., Li Z., Poerschke D.L., Baldwin J.K., Bresnahan B.L., Mara N.A. (2025). Thermal stability of 3D interface Cu/Nb nanolaminates. Scr. Mater..

[26] Spirin A., Zaytsev E., Krutikov V., Paranin S., Khrustov V., Zayats S., Kaygorodov A. (2025). Bulk nanocrystalline Cu-Nb composites produced by powder approach: Processing and characterization. J. Alloys Compd..

[27] Tang C., Duan J., Wan X., Wang D., Sun P., Lai T., Zhang H. (2026). Corrosion behavior and mechanism of laser directed energy deposited CuNb alloys under arc discharge. Int. J. Refract. Met. Hard Mater..

[28] Briffod F., Yasuda K., Zhu J., Shiraiwa T., Jhon M., Gunawan F., Sahay R., Raghavan N., Budiman A.S., Enoki M. (2025). Fatigue and fracture of accumulative roll-bonded Cu/Nb materials: Effects of layer thickness and loading direction. Int. J. Fatigue.

[29] Zhang Y., Gigax J.G., Nizolek T.J., Carpenter J.S., Schneider M.M., Li N., Capolungo L., McCabe R.J. (2022). Tensile and failure behaviors of Cu/Nb nanolaminates: The effects of loading direction, layer thickness, and annealing. Acta Mater..

[30] Zhou J.Q., Hu B., Li B.F., Du Y., Wang J. (2023). Experimental investigation and thermodynamic modeling of Cu-Nb-Si system. Trans. Nonferrous Met. Soc. China.

[31] Cheng J.Y., Radhakrishnan M., Miller C., Mier R., Vogel S.C., Savage D.J., Carpenter J.S., Anderoglu O., Mara N.A. (2023). The influence of thermomechanical treatment pathways on texture and mechanical properties in ARB Cu/Nb nanolaminates. Mater. Sci. Eng. A.

[32] Ni C., Ding C., Wang F., Li H., Zhu Q., Shan D. (2025). Effects of electric current on the mechanical properties of Cu/Nb multilayer composites by accumulative roll bonding. Materials.

[33] Višniakov N., Beinoras P., Kapustynskyi O. (2025). Upset resistance welding of a microcomposite Cu-Nb conductor for pulsed power applications. Metals.

[34] Du Z., Guo C., Li C., Cui J., Ren X. (2024). Experimental investigation of isothermal sections and thermodynamic modeling on the Cu-Nb-Ni system. J. Phase Equilib. Diffus..

[35] Paul M., Alshammari Y., Yang F., Bolzoni L. (2023). Processing and properties of powder metallurgy Ti-Cu-Nb alloys. J. Alloys Compd..

[36] Tang C., Wang D., Sun P., Lai T., Zhang H., Zhou J., Ren G. (2025). The CuNb alloys prepared by laser directed energy deposition: Effect of Ti addition on microstructure and properties. Int. J. Refract. Met. Hard Mater..

[37] Yang Z., Deng F., Tao Z., Yan S., Ma H., Qian M., He W., Zhang Z., Liu Y., Wang L. (2023). Effects on the Microstructure Evolution and Properties of Graphene/Copper Composite during Rolling Process. Materials.

[38] Zhang X., Gao J., Zhang L., Chen Y., Zhang Y., Zhang K. (2024). Molecular Dynamics Study on the Sintering Mechanism and Tensile Properties of Novel Cu Nanoparticle/Graphene Nanoplatelet Composite Solder Paste. Materials.

[39] Xiu Z., Ju B., Zhan J., Zhang N., Wang Z., Mei Y., Liu J., Feng Y., Guo Y., Kang P. (2022). Microstructure Evolution of Graphene and the Corresponding Effect on the Mechanical/Electrical Properties of Graphene/Cu Composite during Rolling Treatment. Materials.

[40] Guan L., Zhang Y., Cheng K., Bai S., Gao Q., Zhang X., Wang X., Li M., Zhao J., Suo J. (2024). Influence of graphite on tribological properties of SiC/Cu/Gr composites with SiO_2_-Cu_2_O glass boundary. Ceram. Int..

[41] Yu J., Wang L., Guan Y., Shao B., Liu Z., Zong Y. (2023). A high strength and high electrical conductivity graphene/Cu composite with good high-temperature stability. Mater. Charact..

[42] Xiao Z., Chen R., Zhu X., Li Z., Xu G., Jia Y., Zhang Y. (2020). Microstructure, and physical and mechanical properties of copper-graphite composites obtained by in situ reaction method. J. Mater. Eng. Perform..

[43] Bident A., Grosseau-Poussard J.-L., Delange F., Addad A., Ji G., Lu Y., Bobet J.-L., Veillere A., Silvain J.-F. (2024). Raman spectroscopy and microstructural characterization of hot-rolled copper/graphene composite materials. Inorganics.

[44] Shi L., Liu M., Yang Y., Liu R., Zhang W., Zheng Q., Ren Z. (2021). Achieving high strength and ductility in copper matrix composites with graphene network. Mater. Sci. Eng. A.

[45] Zhang X., Guo X., Song K., Wang X., Feng J., Li S., Lin H. (2021). Simulation and verification of thermal conductivity of CuCr30 contact material based on morphological changes of Cr particles. Mater. Today Commun..

[46] Liang S., Wang X., Wang L., Cao W., Fang Z. (2012). Fabrication of CuW pseudo alloy by W-CuO nanopowders. J. Alloys Compd..

[47] Cheng Z., Sun Y., Liang Y., Yang S., Seveno D., Guo M. (2025). High-temperature dissolutive wetting: Role of solutal Marangoni convection in multiphase transport. Chem. Eng. J..

[48] Chen B., Hamada S., Li W., Noguchi H. (2023). Crystal plasticity FEM study of material and mechanical effects on damage accumulation mode of fatigue crack propagation. Int. J. Fatigue.

[49] Fang C., Wang F., Wang Z., Lu H., Wang J., Wen K., Xu C., Zhan Q., Li J. (2025). Synergistic optimization of erosion resistance and comprehensive mechanical properties: Designing of Bi-enhanced Ag-SnO_2_ contacts with a neuron cell-like structure. Mater. Today Commun..

